# Far From Help: Exploring the Influence of Regional and Remote Residence on Coastal Visitation and Participation, Risk Perception and Safety Knowledge and Practices

**DOI:** 10.1111/ajr.70018

**Published:** 2025-02-24

**Authors:** Ella G. Pratt, Amy E. Peden, Jasmin C. Lawes

**Affiliations:** ^1^ UNSW Medicine and Health UNSW Sydney Sydney New South Wales Australia; ^2^ School of Population Health, UNSW Sydney Sydney New South Wales Australia; ^3^ UNSW Beach Safety Research Group UNSW Sydney Sydney New South Wales Australia; ^4^ Surf Life Saving Australia Sydney New South Wales Australia

## Abstract

**Objective:**

To explore how self‐reported coastal visitation, participation, risk perception, safety knowledge, experiences and safety practices differed between regional and remote, and major city residents to inform remoteness‐specific coastal drowning prevention efforts.

**Methods:**

This study used data obtained from the annual National Coastal Safety Survey from 2018 to 2023. Data were postweighted proportionally according to age, gender and Australian Statistical Geography Standard classification using 2021 census population data. Descriptive statistics and chi‐square analyses were used to identify key differences in the behaviours, knowledge, and experiences of regional and remote respondents as compared to major city respondents.

**Results:**

A total of 14 210 respondents were included in this dataset. Regional and remote respondents were more likely to visit an unpatrolled beach, particularly when swimming/wading (*n* = 929, 42.3%; χ^2^ = 502.14, *p* < 0.001). However, similar proportions of regional and remote and major city respondents reported following safety practices on patrolled beaches. Regional and remote respondents were more likely to consider sea and weather conditions for all coastal activities, but there was little difference between remoteness groups regarding rip current identification, perceptions and experiences.

**Discussion:**

This study demonstrates that future coastal safety research should consider the impact of remoteness to corroborate the findings of this study: that a significant contributor to the regional and remote drowning burden is a lack of access to patrolled beaches.

**Conclusions:**

Regional and remote residents are more likely to frequent unpatrolled beaches but, if able to visit a patrolled beach, are just as likely as major city residents to adhere to safety practices.

**Summary**

- What is already known on this subject?
  ○ Drowning risk is determined by the interaction of individual behaviours, perceptions, safety knowledge and hazard awareness.
  ○ Regional and remote residents are disproportionately represented in Australian coastal drowning statistics.
  ○ There are limited studies that investigate the impact of residence on the behaviours, attitudes and experiences of Australians towards coastal safety.
- What does this paper add?
  ○ The data used in this study, from the National Coastal Safety Survey, are nationally representative, of a large sample size and span the years 2018 to 2023. Prior to the commencement of this survey, there was no such data that addressed self‐reported coastal safety behaviours and attitudes on such a large scale.
  ○ Using these data, this paper identifies the key differences in coastal visitation and participation, risk perception and safety knowledge and practices between regional and remote residents and those who reside in major cities to inform future coastal safety efforts.

## 1 Introduction

Regional and remote Australians experience significantly poorer health outcomes compared to Australians living in major cities [1]. Drowning is a significant contributor to the high rate of injury‐related morbidity and mortality in regional and remote Australia, and is associated with significant financial, physical and social consequences [2, 3].

In Australia, coastal drowning is a leading contributor to Australia's drowning toll, with 125 coastal drowning deaths recorded in the fiscal period from 2022 to 2023, representing 44% of the total drowning burden [4]. Regional and remote residents are overrepresented in drowning statistics, including those that occur at the coast [5].

Drowning risk is a complex interaction of individual behaviours, safety knowledge, and hazard awareness. Identifying specific demographics that are more likely to adopt risky behaviours on the coast, due to knowledge deficits or other characteristics like gender, age or familiarity with coastal hazards, is key to developing effective safety strategies [6].

While there have been advancements in our understanding of the particular behaviours and attitudes that put individuals at greater risk of coastal drowning [7, 8, 9], there is a limited investigation into regional and remote residents specifically. This is in spite of the disproportionate representation of regional and remote individuals in drowning statistics [6]. With more individuals moving from the city to regional and remote areas [10], and coastal drowning fatalities remaining concerningly high [3], further research is required to understand if and how the behaviours, attitudes and experiences of regional and remote individuals towards coastal safety differ from those of individuals residing in major cities. This will aid in the development of coastal drowning prevention strategies for regional and remote Australians.

This study used data from the National Coastal Safety Survey (NCSS), a Surf Life Saving Australia (SLSA) initiative conducted annually, to explore how regional and remote residents in Australia compare to major city residents in self‐reported coastal visitation, participation, risk perception, coastal safety knowledge and experiences and safety practices.

Data are nationally representative, of a large sample size and span the years 2018–2023. Prior to the commencement of the NCSS, there were no available data that addressed self‐reported behaviours and attitudes towards coastal safety on such a large scale. These data are valuable in that they effectively target a knowledge gap in Australian coastal safety research and help to inform future coastal safety initiatives, particularly those aimed at regional and remote Australians [11].

This study aimed to answer the following research questions:

1. How do regional and remote residents in Australia compare to major city residents in self‐reported:
  - Coastal visitation,
  - Participation,
  - Risk perception,
  - Coastal safety knowledge and experiences and
  - Safety practices.
2. Do risk‐taking behavioural tendencies or perceptions differ from reported safety practices by remoteness?
3. Using the differences highlighted in RQ1 and RQ2, can a coastal drowning risk profile be developed for regional and remote Australians?

By addressing these research questions, this study will identify differences that may exist between the behaviours and aptitudes of regional and remote, and major city Australians towards the coast. It is hoped that this will help inform policymakers in developing specific and effective strategies to reduce the coastal drowning burden for regional and remote Australians, who remain disproportionately represented in national drowning statistics [5].

## 2 Methodology

### 2.1 Study Design

This retrospective cross‐sectional total population representative study was completed using a merged data set of NCSS responses captured in the period spanning 2018–2023. The survey questions were developed independently by OmniPoll, commissioned by SLSA, and answered by participants recruited by Lightspeed. Questions were categorised into five groups for ease of data analysis: coastal visitation, participation, risk perception, coastal safety knowledge and experiences and safety practices.

### 2.2 Question Types

The structure and format of NCSS has previously been described in great detail [11], but in brief the survey contained multiple question types (including open text, four‐digit responses and categorical questions) that span from general themes, such as demographics, visitation and hazard perception, to more specific topics, such as coastal activity participation. Respondents are screened early in the questionnaire so that specific questions are only offered to relevant respondents. Questions and themes are revised annually, and additional questions are added to increase the scope and size of NCSS.

### 2.3 Data Collection

NCSS was conducted online over the years 2018–2023. Initial email invitations were sent to panel members over the age of 16 according to predetermined demographic quotas. Demographic quotas are determined using the triple interlocking quota sampling method, combining age, gender and geographical area.

Participants receive financial remuneration for participating by earning points which can be reimbursed as a deposit or to purchase vouchers. NCSS data have been provided to SLSA in a deidentified format to maintain participant anonymity.

### 2.4 Data Analysis

Survey responses were exported to Excel for cleaning and reformatting. Using data from the 2021 census, postcodes provided by respondents were matched to an Australian Statistical Geographical Standard (ASGS) classification [12]. For postcodes associated with two or more ASGS classifications, the classification with the greater population was used. Respondents who provided incorrect postcodes, postcodes for PO boxes or no postcode were excluded from the study (*n* = 216).

Data were weighted according to age, gender and ASGS classification using the 2021 census population data [12] and were categorised into two separate groups according to ASGS classification: major city and regional and remote (combining inner regional, outer regional, remote and very remote classifications). Following data cleaning, reformatting and weighting, there were 14 210 responses matched to ASGS remoteness classifications.

Data analysis was conducted using SPSS (version 27.0.1.0). Descriptive statistics and chi‐square analyses were used to explore the data and determine categorical differences. Statistical significance was deemed *p* < 0.05. Statistically significant results are represented in bold text.

## 3 Results

### 3.1 Study Demographics

A total of 14210 responses were included in this dataset. For major city respondents, 34.3% (*n* = 3209) lived < 10 km and 29.3% (*n* = 2745) lived 10–25 km from the coast. While 36.7% (*n* = 1778) of regional and remote respondents lived < 10 km from the coast, 29.1% (*n* = 1412) lived over 100 km from the coast. The most common age group for major city (*n* = 4497, 48.1%) and regional and remote (*n* = 2701, 55.7%) remoteness groups was 35–49 and 50–64 (Table 1).

**TABLE 1:** 2018–2023 NCSS responses by remoteness and sex, age group and distance from the coast (*n* = 14 210).

Total		Remoteness				Test statistic (*p*)
		Major cities		Regional/Remote		
		*N*	%	*N*	%	
		9361	100	4849	100	
Gender	Male	4608	49.2	2327	48.0	χ^2^ = 1.96 (*p* > 0.05)
	Female	4753	50.8	2522	52.0	
Age group (years)	16–24	1364	14.6	449	9.3	χ^2^ = 264.73 (***p* < 0.001**)
	25–34	1780	19.0	630	13.0	
	35–49	2432	26.0	1197	24.7	
	50–64	2065	22.1	1504	31.0	
	65–69	681	7.3	427	8.8	
	70+	1040	11.1	641	13.2	
Distance from the coast	< 10 km	3209	34.3	1778	36.7	χ^2^ = 2074.01 (***p* < 0.001**)
	10–25 km	2745	29.3	532	11.0	
	26–50 km	1784	19.1	463	9.6	
	51–100 km	758	8.1	521	10.7	
	Over 100 km	479	5.1	1412	29.1	
	Can't say	386	4.1	143	2.9	

*Note:* Significant chi‐square analyses (*p* < 0.05) are presented in bold.

### 3.2 Coastal Visitation

The frequency of coastal visits between the two remoteness groups was similar, with 24.7% (*n* = 2317) of major city respondents visiting the coast 3–11 times per year, compared to 18.3% (*n* = 888) of regional and remote respondents. Regional and remote respondents were significantly less likely to visit the coast (1–2 times per year) (χ^2^ = 261.20, *p* < 0.001). However, regional and remote respondents (*n* = 929, 42.3%) were significantly more likely to visit unpatrolled beaches for swimming/wading (*n* = 887, 18.6%; χ^2^ = 502.14, *p* < 0.001). Regional and remote respondents were also more likely to visit an unpatrolled beach or coastline when surfing or bodyboarding, using watercraft or jet skis, scuba diving or snorkelling (Table 2).

**TABLE 2:** Coastal locations most likely to be chosen for popular coastal activities by remoteness.

Activity	Location	Remoteness				Test statistic (*p*)
		Major cities		Regional/Remote		
		%	*N*	%	*N*	
Swimming or wading	Patrolled beach during patrol hours only	44.5	2117	25.3	556	χ^2^ = 502.14 (***p* < 0.001**)
	Patrolled beach, but not always during patrolled hours	26.0	1238	19.9	436	
	Unpatrolled beach	18.6	887	42.3	929	
Surfing or bodyboarding	Patrolled beach during patrol hours only	44.5	2117	25.3	556	χ^2^ = 41.27 (***p* < 0.001**)
	Patrolled beach, but not always during patrolled hours	26.0	1238	19.9	436	
	Unpatrolled beach	18.6	887	42.3	929	
Watercraft	Patrolled beach during patrolled hours only	18.0	143	8.3	32	χ^2^ = 48.30 (***p* < 0.001**)
	Patrolled beach, but not always during patrolled hours	15.4	123	8.9	34	
	Unpatrolled beach	11.6	93	21.8	83	
Snorkelling	Patrolled beach during patrolled hours only	26.3	258	9.7	42	χ^2^ = 64.67 (***p* < 0.001**)
	Patrolled beach, but not always during patrolled hours	9.8	96	8.0	34	
	Unpatrolled beach or coastline	24.1	236	39.0	167	
Scuba diving	Patrolled beach during patrolled hours only	21.0	46	8.4	8	χ^2^ = 21.94 (***p* = 0.001**)
	Patrolled beach, but not always during patrolled hours	11.0	24	3.4	3	
	Unpatrolled beach or coastline	12.1	26	15.0	13	
Jet ski/personal watercraft	Patrolled beach during patrolled hours only	27.7	105	16.5	28	χ^2^ = 26.11 (***p* < 0.001**)
	Patrolled beach, but not always during patrolled hours	9.0	34	3.8	6	
	Unpatrolled beach	7.9	30	10.1	17	

*Note:* Significant chi‐square analyses (*p* < 0.05) are presented in bold.

A larger proportion of major city respondents reported visiting patrolled beaches during patrolled hours for all activities, particularly swimming/wading (*n* = 2117, 44.5%) and surfing or bodyboarding (*n* = 301, 43.4%). While a bigger proportion of major city respondents also reported visiting patrolled beaches but not always during patrolled hours for these activities, the difference between the two remoteness groups was notably smaller (Table 2).

Fishing (χ^2^ = 16.02, *p* = 0.001) and sailing/boating locations (χ^2^ = 47.12, *p* < 0.001) were similar between remoteness groups, the most notable difference being that 42% (*n* = 55) of MC respondents sail/boat at a bay or harbour, compared to 29.4% (*n* = 208) of RR respondents (Table S1).

### 3.3 Participation

When choosing a location for swimming/wading, only 29.5% (*n* = 646) of regional and remote respondents considered lifeguarding or lifesaving service patrols important, compared to 40.0% (*n* = 1906) of major city respondents (χ^2^ = 73.46, *p* < 0.001). This was similarly observed in respondents choosing a surfing or bodyboarding location, with 23.8% (*n* = 65) of regional and remote respondents considering patrolling an important factor, compared to 35.3% (*n* = 245) of major city respondents (χ^2^ = 12.32, *p* < 0.001).

**FIGURE 1:**
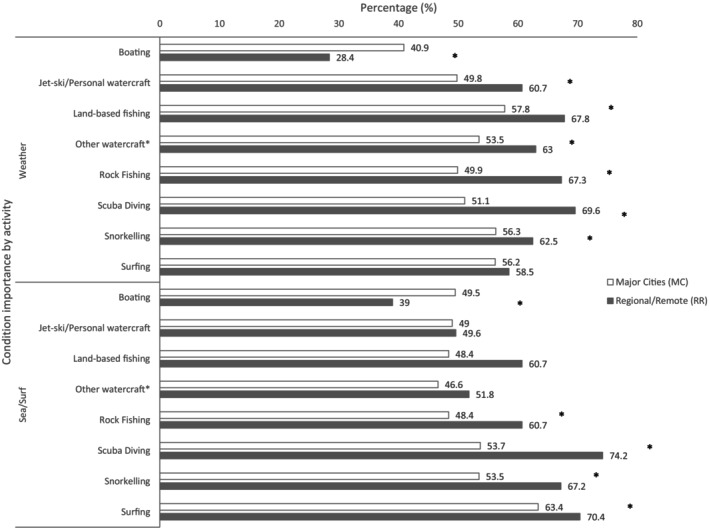
Proportion of respondents that considered weather or sea/surf conditions important by coastal activity and remoteness. Significant chi‐square analyses (*p* < 0.05) are denoted by *. *Other watercraft include paddle craft, stand‐up paddle, paddle boarding, kite surfing and wind surfing.

Major city respondents were more concerned about the presence of lifeguards or lifesaving services when choosing a location for coastal activities, but regional and remote respondents were more likely to consider sea or weather conditions important. In total, 71.6% of regional and remote respondents (*n* = 580) considered the weather conditions an important consideration when choosing a boating location, compared to 58.9% (*n* = 760) of major city respondents (χ^2^ = 32.85, *p* < 0.001). Likewise, 61.1% (*n* = 497) of regional and remote respondents considered sea conditions important, compared to 50.3% (*n* = 649) of major city respondents (χ^2^ = 23.38, p < 0.001). This trend is also evident across other coastal activities (Figure 1).

Just over a third (35.7%, *n* = 207) of regional and remote respondents fish every time they go boating, compared to 18.0% (*n* = 181) of major city respondents. 17.1% (*n* = 171) of major city respondents never fish when they go boating, compared to 5.7% (*n* = 33) of regional and remote respondents (χ^2^ = 92.88, *p* < 0.001).

### 3.4 Risk Perception

Major city and regional and remote respondents displayed similar attitudes and behaviours towards coastal hazards. Almost half of the major city (48.6%, *n* = 4552) and regional and remote (47.1%, *n* = 2282) respondents considered the coast to be somewhat hazardous (χ^2^ = 44.33, *p* < 0.001). Similarly, 42.9% (*n* = 4013) of major city and 44% (*n* = 2131) of regional and remote respondents consider the beach to be somewhat hazardous (χ^2^ = 40.74, *p* < 0.001). This was also observed in respondents' perception of different coastal activities, with the most common answer for both remoteness groups being ‘somewhat hazardous’ (Table S2).

In total, 43.8% (*n* = 4090) of major city and 47.1% (*n* = 2284) of regional and remote respondents considered rip currents (rips) to be ‘extremely hazardous’ (χ^2^ = 33.82, *p* < 0.001). However, 32.5% (*n* = 2047) of major city and 33.0% (*n* = 979) of regional and remote respondents were only ‘somewhat concerned’ about being unintentionally caught in a rip, the most popular response. Major city and regional and remote females were more likely to view rips as ‘extremely hazardous’ than males; they were also more likely to be ‘extremely’ or ‘somewhat’ concerned about getting caught in a rip (χ^2^ = 66.02, *p* < 0.001) (Table S2).

A bigger proportion of regional and remote respondents considered themselves experienced enough to take risks in all coastal activities compared to major city respondents, but the only statistically significant difference was for swimming/wading, rock‐and land‐based fishing. In total, 55.5% (*n* = 1161) of regional and remote respondents perceived themselves as experienced enough to take risks when swimming/wading, compared to 51.8% (*n* = 2365) of major city respondents (Table S2). In both remoteness groups, males and under 50‐year‐old respondents were significantly more likely to consider themselves experienced enough to take risks (χ^2^ = 6.03, *p* = 0.014) when compared to females and other age groups.

Just over half (58.5%, *n* = 148) of regional and remote respondents considered themselves experienced enough to take risks when rock‐fishing, compared to 47.9% (*n* = 169) of major city respondents (χ^2^ = 6.67, *p* = 0.010). Similarly, 58.1% (*n* = 422) of regional and remote respondents considered themselves experienced enough to take risks when land‐based fishing compared to 51.5% (*n* = 500) of major city respondents (Table S2). In both cases, a larger proportion of males than females believed themselves experienced enough to take risks (χ^2^ = 7.08, *p* = 0.008).

### 3.5 Coastal Safety Knowledge and Experience

There was little difference between major city and regional and remote respondents who reported they had been rescued or had rescued someone on the coast. If the respondent was rescued, the time of day and the relationship of the rescuer to the respondent did not differ significantly between remoteness groups. However, 27.8% (*n* = 45) of regional and remote respondents were rescued at an unpatrolled beach compared to 19.2% (*n* = 79) of major city respondents (χ^2^ = 14.21, *p* = 0.048) (Table S3). Regional and remote respondents (*n* = 138, 33.3%) were also more likely to conduct a rescue at an unpatrolled beach compared to major city respondents (*n* = 177, 22.9%).

Regional and remote respondents were more likely to be rescued by someone they knew or a stranger, whereas major city respondents were more likely to be rescued by a lifesaver, but these results were not significant (χ^2^ = 5.18, *p* = 0.159). However, a greater proportion of regional and remote respondents had rescued a stranger (χ^2^ = 10.30, *p* = 0.006).

While similar proportions of regional and remote and major city respondents reported having ever been caught in a rip, a slightly greater proportion of regional and remote respondents reported being caught in a rip at a beach outside of patrol hours (*n* = 207, 21.4%) or at an unpatrolled beach (*n* = 177, 18.2%, χ^2^ = 56.22, *p* < 0.001) (Table S3).

**FIGURE 2:**
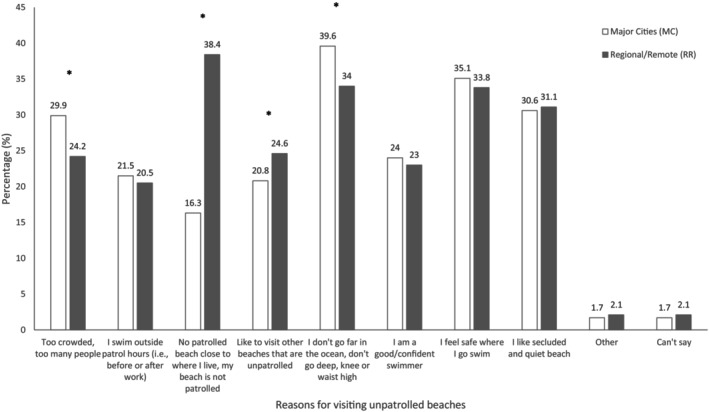
Self‐reported reasons for visiting unpatrolled beaches by remoteness. Significant chi‐square analyses (*p* < 0.05) are denoted by *.

38.2% (*n* = 413) of regional and remote respondents reported a lack of nearby patrolled beaches as a reason for visiting unpatrolled beaches, compared to 16.4% (*n* = 432) of major city respondents (χ^2^ = 207.55, *p* < 0.001). There was little significant difference between the proportions of major city and regional and remote respondents who selected other reasons for visiting unpatrolled beaches (Figure 2).

There was little difference between the sources that major city and regional and remote respondents reported turning to for coastal safety information. Similar proportions of major city (*n* = 1388, 14.8%) and regional and remote respondents (*n* = 835, 17.2%) reported having been exposed to advertising about rips in the past 3 months (χ^2^ = 13.90, *p* < 0.001) (Table S3).

### 3.6 Safety Practices

Regional and remote respondents (*n* = 1039, 21.4%) were more likely to own lifejackets than major city respondents (*n* = 1312, 14.0%; χ^2^ = 127.08, *p* < 0.001). However, major city respondents were more likely to wear lifejackets for almost all coastal activities, particularly when swimming (*n* = 211, 16.1%; χ^2^ = 23.91, *p* < 0.001) and boat‐based fishing (*n* = 453, 34.5%; χ^2^ = 40.52, *p* = 0.002) (Table S4).

Major city respondents were more likely to ‘always’ (*n* = 1772, 37.2%) or ‘most of the time’ (*n* = 1550, 32.6%) swim/wade at a patrolled beach during patrolled times (χ^2^ = 240.14, *p* < 0.001).

However, if on a patrolled beach, there was little difference between remoteness groups swimming/wading between the flags, with the majority ‘always’ doing so (χ^2^ = 76.15, *p* < 0.001). This was also observed in other swimming/wading safety practices at a patrolled beach (Table 3).

**TABLE 3:** How often respondents reported adhering to common swimming or wading safety practices on patrolled beaches by remoteness?

Swimming or wading safety practice	How often safety practice is followed	Remoteness				Test statistic (*p*)
		Major cities		Regional/Remote		
		%	*N*	%	*N*	
Swim or wade at a patrolled beach during patrol times	Always	37.2	1772	29.4	647	χ^2^ = 240.14 (***p* < 0.001**)
	Most of the time	32.6	1550	24.0	528	
	Sometimes	23.9	1140	30.6	672	
	Never	3.6	173	9.7	214	
	Can't say	2.6	126	6.2	137	
Swim or wade between the red and yellow flags when on a patrolled beach	Always	53.1	2530	53.0	1164	χ^2^ = 76.15 (***p* < 0.001**)
	Most of the time	27.1	1291	22.6	496	
	Sometimes	14.5	691	14.2	312	
	Never	3.2	151	5.1	112	
	Can't say	2.0	97	5.2	114	
Check surf conditions with a lifesaver, lifeguard or other authoritative source	Always	23.9	1139	22.1	485	χ^2^ = 27.68 (***p* < 0.001**)
	Most of the time	18.9	900	18.2	400	
	Sometimes	27.7	1318	26.4	580	
	Never	25.2	1198	26.2	575	
	Can't say	4.3	206	7.2	158	
Check for and obey safety signs posted on the beach	Always	58.5	2783	59.0	1297	χ^2^ = 22.55 (***p* < 0.001**)
	Most of the time	26.8	1276	25.0	550	
	Sometimes	11.5	549	10.7	236	
	Never	2.0	94	2.6	56	
	Can't say	1.2	59	2.6	58	
Follow the advice of the local lifesaver or lifeguard when on a patrolled beach	Always	64.9	3091	66.4	1460	χ^2^ = 101.40 (***p* < 0.001**)
	Most of the time	20.8	992	16.9	372	
	Sometimes	9.6	459	6.7	147	
	Never	1.9	93	3.1	69	
	Can't say	2.6	126	6.8	149	

*Note:* Significant chi‐square analyses (*p* < 0.05) are presented in bold.

Similar proportions of regional and remote (*n* = 1101, 50.1%) and major city (*n* = 2138, 44.9%) respondents reported always looking for rips prior to entering the water (χ^2^ = 23.37, *p* < 0.001). However, regional and remote respondents were more likely to always avoid swimming/wading under the influence of alcohol/drugs (*n* = 1567, 71.3%, χ^2^ = 10.96, *p* = 0.027) (Table S4).

Just over half (59.3%, *n* = 147) of regional and remote respondents reported that they always checked for and obeyed safety signs when rock‐fishing, compared to 49.2% (*n* = 184) of major city respondents (χ^2^ = 11.88, *p* = 0.018). However, they were less likely to wear or carry a life jacket or buoyancy aid.

Regional and remote respondents were more likely to ‘always’ follow particular safety practices than major city respondents when boating. Notably, 86.8% (*n* = 706) of regional and remote respondents reported always carrying necessary safety equipment, compared to 78.5% (*n* = 1013) of major city respondents (χ^2^ = 26.15, *p* < 0.001). Accordingly, a greater proportion of regional and remote respondents carried almost all types of safety equipment when boating (Table 4).

**TABLE 4:** Safety practices and equipment carried when boating by remoteness.

Boating safety practices and equipment		Remoteness				Test statistic (*p*)
		Major cities		Regional/Remote		
		%	*N*	%	*N*	
How often do you carry necessary safety equipment when boating?	Always	78.5	1013	86.8	706	χ^2^ = 26.15 (***p* < 0.001**)
	Most of the time	12.8	165	8.7	70	
	Sometimes	5.5	71	2.7	22	
	Never	0.5	7	0.7	5	
	Can't say	2.6	33	1.1	9	
Equipment carried when boating	Flares	60.1	763	74.1	596	χ^2^ = 42.74 (***p* < 0.001**)
	Mobile phone	87.5	1111	92.8	747	χ^2^ = 14.86 (***p* < 0.001**)
	Radio	61.9	786	68.3	550	χ^2^ = 8.89 (***p* = 0.003**)
	Anchor	74.1	941	86.5	696	χ^2^ = 45.24 (***p* < 0.001**)
	Torch	66.2	841	76.1	613	χ^2^ = 23/16 (***p* < 0.001**)
	Bucket	63.2	802	82.4	663	χ^2^ = 87.61 (***p* < 0.001**)
	Lifejacket	89.9	1141	92.5	744	χ^2^ = 4.32 (***p* = 0.038**)
	Buoyancy aid	56.7	720	58.8	473	χ^2^ = 0.88 (*p* = 0.347)
	EPIRBs (Emergency Position‐Indicating Radio Beacons)	42.9	424	63.4	364	χ^2^ = 61.32 (***p* < 0.001**)

*Note:* Significant chi‐square analyses (*p* < 0.05) are presented in bold.

## 4 Discussion

It is well understood that regional and remote Australians are disproportionately represented in Australian drowning statistics and that coastal drowning significantly contributes to Australia's overall drowning toll [6, 10, 13, 14]. Individual behaviours, attitudes, knowledge and awareness inform coastal safety practices and accordingly influence coastal drowning risk.

This study identified the primary differences between the self‐reported coastal attitudes, knowledge, behaviours and practices of regional and remote and major city Australians by answering the proposed research questions. Notably, regional and remote respondents were more likely to consider weather and sea/surf conditions prior to visiting the coast and were much more likely to visit, conduct a rescue, or be rescued at an unpatrolled beach (RQ1). Both remoteness groups demonstrated similar risk‐taking behavioural tendencies if on a patrolled beach and similar attitudes towards various coastal hazards. However, regional and remote respondents were much more likely to forgo using lifejackets and visit unpatrolled beaches (RQ2). Given these differences, a typology of regional and remote drowning risk can be developed (RQ3) to inform future prevention strategies (see Practical Applications).

### 4.1 Unpatrolled Beaches

Regional and remote respondents were more likely to visit unpatrolled beaches for all coastal activities, yet the only significant difference between the two remoteness groups was the lack of patrolled beaches near regional and remote respondents' residences. If at a patrolled beach, similar proportions of major city and regional and remote respondents reported following safety practices, such as always swimming between the flags.

In Australia, the most effective strategy to ensure beachgoer safety is through the provision of lifeguard services and the use of beach safety flags [15]. Accordingly, most coastal drowning deaths occur at unpatrolled beaches, which account for the majority of Australia's beaches [2]. Prior studies surveying beachgoers have suggested that location and ease of access are the primary determinants of beach visitation [16, 17]. However, these studies have a strong representation of domestic tourists, who may be more likely to frequent an unpatrolled beach due to their proximity to coastal tourist accomodation [18].

The results of this study suggest that while regional and remote respondents were more likely to visit an unpatrolled beach, regional and remote and major city respondents did not differ significantly in their approach to coastal safety if on a patrolled beach. Prior research indicates that inland regional and remote residents are very likely to swim between the flags at patrolled beaches; however, this study did not consider coastal regional and remote residents [16]. Other research has suggested that unpatrolled beach visitors have poor rip identification skills (Australia's number one coastal hazard [3]) and do not observe safety signage [17]. However, it cannot be assumed that these unpatrolled beach visitors are regional and remote residents, due to the high proportion of domestic visitors/tourists in the study population [16, 17]. Further research needs to be conducted in coastal areas with less tourist presence to determine the relationship between regional and remote Australians, unpatrolled beaches and safety practices, to corroborate the findings of this study.

Given that coastal locations are the most common drowning locations for regional and remote Australians [16], and most drownings occur at unpatrolled beaches [3], the bystander, emergency and acute healthcare response is significant for reducing drowning‐related mortality and morbidity. While patrolled beaches are essential to reducing coastal drowning, the majority of surf lifesavers are volunteers [2], and this makes sourcing and training lifesavers in smaller regional and remote Australian communities challenging. This is particularly relevant in light of structural population ageing in regional and remote communities, which increases demand but reduces supply for volunteer lifesavers [19]. In addition to extending lifesaving service delivery to high‐risk regional and remote locations, continuing to support normative or experiential educational‐based initiatives in rural communities, such as the Beach to Bush or lifeguard learning programs, can help promote safer behaviours [20]. In situ safety measures such as signage and the deployment of technological‐based tools such as drones and emergency response beacons [21] are also an important component of the emergency response network for unpatrolled coastal locations.

Further, regional and remote Australians have poorer access to healthcare and emergency services [22, 23, 24]. The quality of regional and remote healthcare, therefore, may contribute to the high burden of Australian coastal drownings, but further research is required to better understand this relationship.

### 4.2 Environmental Safety Hazard Practices

While regional and remote respondents were more likely to consider weather and sea conditions important determinants when choosing a location for coastal activities, both remoteness groups demonstrated similar behaviours, knowledge and experiences towards rips, with most considering rips ‘extremely hazardous’.

Rips are the dominant hazard found along the Australian coast [3] and are increasingly explored in studies examining the characteristics of coastal visitors. Previous literature has identified that beachgoers with basic knowledge about rips are more likely to avoid swimming in rips [25], but also that beachgoers are likely overconfident in their rip identification skills [25, 26, 27]. There is, however, limited investigation into how rip knowledge and behaviours differ between remoteness groups. One study identified that regional and remote inland Australians and Australian beachgoers did not differ in rip identification or the likelihood of swimming in a rip but failed to specifically explore regional and remote coastal residents [16]. The extent of rip safety strategies and awareness campaigns in Australia could explain the similar behaviours and attitudes demonstrated between remoteness groups in this study [28].

Regional and remote Australians demonstrated a significantly higher consideration for weather and sea conditions compared to their major city counterparts. This could be explained by regional and remote Australians having to take greater ownership of their own safety in the absence of coastal patrolling services. While there is extensive research that addresses the impact of physical coastal hazards on drowning [29, 30], there is limited literature that focuses on individual behaviours, attitudes, knowledge and awareness of weather and sea conditions. One study identified beach user preferences for both weather and ocean conditions on three Australian tourist beaches but failed to investigate how behaviours and attitudes differed between visitors [31].

### 4.3 Bystander Rescues

Bystander rescuers are members of the public who assist someone in distress and often represent the only immediate form of rescue available at unpatrolled locations. Tragically, these bystanders often drown while attempting rescues. Hence, bystander rescues have become an increasingly important issue in drowning prevention research [32]. This study developed a bystander rescue/rescue typology by identifying that regional and remote respondents are not only more likely to visit an unpatrolled beach, but they are also more likely to be rescued or conduct a rescue at an unpatrolled beach.

Despite the hazards associated with rescuing someone who is drowning, bystanders are often ill‐prepared to attempt a rescue [33]. Various studies have characterised common locations for bystander rescues, with most occurring more than 1 km from the nearest lifesaving service and in regional and remote areas. However, these studies fail to identify the characteristics of the bystander or the rescued individual [5, 32, 34]. Further research is required to understand whether regional and remote individuals are more at risk of drowning when conducting a bystander rescue, or whether it is unpatrolled, regional and remote coastal locations that put bystanders at risk. If certain individual characteristics, such as residence, increase the risk of a bystander rescue occurring, specific education strategies can be developed to increase the likelihood of successful rescue and resuscitation, as well as reducing the fatal bystander rescuer drowning toll [34].

### 4.4 Lifejackets

This study highlighted inconsistencies in regional and remote respondents' risk perception and lifejacket use, a recommended drowning risk reduction strategy [35]. A larger proportion of regional and remote respondents considered themselves experienced enough to take risks across several coastal activities, particularly rock‐fishing. Accordingly, they were more likely to obey safety practices, except for wearing a lifejacket or buoyancy aid. Further, regional and remote respondents were more likely to own lifejackets, but major city respondents were more likely to report wearing them across all coastal activities.

Multiple studies have accounted for the efficacy of lifejackets in drowning prevention [35]. Despite this, individuals may choose not to wear lifejackets when on the coast, associating them with swimming inability or boating inexperience [36, 37]. While one study has investigated factors associated with increased and decreased lifejacket use, there is no evidence that considers this relationship with respect to remoteness [38].

The difference in reported lifejacket use between regional and remote and major city respondents may be related to legislation in local Australian governments mandating the use of lifejackets for rock‐fishing [39] and boating [40]. Regional and remote residents may be less aware of the laws surrounding lifejacket use or less likely to adhere to laws with less enforcement in regional and remote areas. However, the larger proportion of regional and remote respondents who report owning lifejackets could represent their need to take responsibility for their own safety in the absence of patrolling services. This relationship requires further research to ensure best practice in legislation development, law enforcement and drowning prevention.

### 4.5 Strengths and Limitations

This study makes a valuable contribution to the existing literature by considering the differences between remoteness groups, addressing a significant knowledge gap relating to our understanding of coastal visitation, participation, risk perception, safety knowledge, experiences and practices, which were often missing from other studies [5, 17, 25, 31, 32, 34, 38].

However, despite the novelty of this research, there were several limitations. This study focused solely on respondent remoteness and did not consider the impact of distance to the coast. Further, in the absence of respondents providing their ASGS classification, postcodes were used to categorise respondents according to their remoteness classification. If a postcode belonged to two or more ASGS classifications, the classification with the greater population was used, potentially artificially inflating the proportion of respondents from more populous remoteness classifications. This may not accurately represent the proportion of the respondents who were from major city or regional and remote areas.

A final limitation that should be acknowledged is that this study utilised survey data, which can be prone to recall bias as respondents' answers are dependent on their ability to recall and report past events [11]. However, the impacts of social desirability bias are mitigated by the anonymous nature of the survey [11].

### 4.6 Practical Applications

Coastal drowning continues to be a topic of national importance; understanding the behaviours, attitudes, awareness and knowledge that underlie different groups is key when developing tailored interventions. This study has developed a typology of regional and remote drowning risk by identifying the increased likelihood that they will visit unpatrolled coastal locations and consider environmental safety hazard practices, but often opt out of lifejacket use. While further research is required to corroborate these findings, this study has identified key features of the relationship between regional and remote Australians and the coast. This is fundamental for future prevention strategies, campaigns, legislation and education tools.

Further, this study supports the provision of additional resources and funds towards surf lifesaving services in regional and remote Australia. This is particularly evident when considering how much more likely regional and remote respondents were to visit an unpatrolled beach, yet how little they differed from major city respondents in reported safety practices when on a patrolled beach. This merely reinforces the greater issue affecting regional and remote Australians: poorer access to healthcare and preventative resources [22, 23, 24].

## 5 Conclusions

This study found that regional and remote respondents were more likely to visit unpatrolled beaches and less likely to wear lifejackets for coastal activities but did not differ in their likeliness to adhere to safety practices on patrolled beaches or in their rip identification skills. Additionally, regional and remote respondents were more attuned to sea and weather conditions. Further research needs to be dedicated to understanding the impact of remoteness on bystander rescues, lifejacket use and the characteristics of unpatrolled beach visitors. Overall, this study strengthens the argument for more comprehensive lifesaving services in regional and remote Australia and reinforces the importance of tailored prevention strategies for different population groups against avoidable coastal drowning deaths.

## Author Contributions

**Ella G. Pratt:** conceptualization, writing – original draft, methodology, writing – review and editing, investigation, validation, visualization. **Amy E. Peden:** supervision, resources, writing – review and editing. **Jasmin C. Lawes:** supervision, resources, writing – review and editing, software.

## Ethics Statement

This project and survey data has been approved for use by UNSW Sydney Human Research Ethics Committee Panel B: Arts, Humanities & Law (HC200950).

## Supporting information


**Table S1.** Fishing and sailing/boating locations by remoteness.
**Table S2.** Self‐reported risk perception to the coast and coastal activities by remoteness.
**Table S3.** Self‐reported coastal safety knowledge and experiences by remoteness.
**Table S4.** Self‐reported safety practices and equipment by coastal activity and remoteness.

## Data Availability

Research data are not shared.

## References

[1] D. H. Taylor , A. E. Peden , and R. C. Franklin , “Disadvantaged by More Than Distance: A Systematic Literature Review of Injury in Rural Australia,” Safety 8, no. 3 (2022): 66.

[2] A. E. Peden , A. J. Mahony , P. D. Barnsley , and J. Scarr , “Understanding the Full Burden of Drowning: A Retrospective, Cross‐Sectional Analysis of Fatal and Non‐Fatal Drowning in Australia,” BMJ Open 8, no. 11 (2018): e024868. 10.1136/bmjopen-2018-024868 PMC6254411 30473541

[3] Surf Life Saving Australia , National Coastal Safety Report 2022 (Surf Life Saving Australia, 2022).

[4] Royal Life Saving Society ‐ Australia , National Drowning Report 2023 (Royal Life Saving Society ‐ Australia, 2023).

[5] R. C. Franklin , A. E. Peden , R. W. Brander , and P. A. Leggat , “Who Rescues Who? Understanding Aquatic Rescues in Australia Using Coronial Data and a Survey,” Australian and New Zealand Journal of Public Health 43, no. 5 (2019): 477–483, 10.1111/1753-6405.12900. 31180612

[6] D. H. Taylor , A. E. Peden , and R. C. Franklin , “Next Steps for Drowning Prevention in Rural and Remote Australia: A Systematic Review of the Literature,” Australian Journal of Rural Health 28, no. 6 (2020): 530–542, 10.1111/ajr.12674. 33215761

[7] M. Woods , W. Koon , and R. W. Brander , “Identifying Risk Factors and Implications for Beach Drowning Prevention Amongst an Australian Multicultural Community,” PLoS One 17, no. 1 (2022): e0262175, 10.1371/journal.pone.0262175. 35015768 PMC8751996

[8] M. Abercromby , G. Crawford , L. Nimmo , and J. E. Leavy , “‘I Never Had a Thought About Drowning’. Exploring Water Safety Attitudes and Practices Among Older Adults in Western Australia,” Health Promotion Journal of Australia 33, no. 2 (2022): 524–532, 10.1002/hpja.522. 34278642

[9] R. Birch , D. Morgan , J. Arch , and B. Matthews , “Rock Fisher Behaviours and Perceptions Regarding Drowning Risk Assessed by Direct Observation and Self‐Report: A Public Awareness Campaign Evaluation,” Health Promotion Journal of Australia 33, no. 1 (2022): 399–409, 10.1002/hpja.583. 35179816 PMC9790509

[10] J. C. Lawes , L. Strasiotto , S. Daw , and A. E. Peden , “When Natural Hazards Intersect With Public Health: A Preliminary Exploration of the Impact of Bushfires and the COVID‐19 Pandemic on Australian Coastal Drowning Fatalities,” International Journal of Environmental Research and Public Health 18, no. 10 (2021): 5314. 34067693 10.3390/ijerph18105314 PMC8156160

[11] J. C. Lawes , L. Uebelhoer , W. Koon , et al., “Understanding a Population: A Methodology for a Population‐Based Coastal Safety Survey,” PLoS One 16, no. 8 (2021): e0256202, 10.1371/journal.pone.0256202. 34388222 PMC8362993

[12] Australia Bureau of Statistics , “Remoteness Structure,” 2021, https://www.abs.gov.au/statistics/statistical‐geography/remoteness‐structure.

[13] S. M. Willcox‐Pidgeon , R. C. Franklin , P. A. Leggat , and S. Devine , “Identifying a Gap in Drowning Prevention: High‐Risk Populations,” Injury Prevention 26, no. 3 (2020): 279–288, 10.1136/injuryprev-2019-043432. 31907207 PMC7279566

[14] W. Koon , A. Peden , J. C. Lawes , and R. W. Brander , “Coastal Drowning: A Scoping Review of Burden, Risk Factors, and Prevention Strategies,” PLoS One 16, no. 2 (2021): e0246034, 10.1371/journal.pone.0246034. 33524054 PMC7850505

[15] K. M. White and M. K. Hyde , “Swimming Between the Flags: A Preliminary Exploration of the Influences on Australians' Intentions to Swim Between the Flags at Patrolled Beaches,” Accident Analysis and Prevention 42, no. 6 (2010): 1831–1838, 10.1016/j.aap.2010.05.004. 20728634

[16] A. Williamson , J. Hatfield , S. Sherker , R. Brander , and A. Hayen , “A Comparison of Attitudes and Knowledge of Beach Safety in Australia for Beachgoers, Rural Residents and International Tourists,” Australian and New Zealand Journal of Public Health 36, no. 4 (2012): 385–391, 10.1111/j.1753-6405.2012.00888.x.

[17] L. Uebelhoer , W. Koon , M. D. Harley , J. C. Lawes , and R. W. Brander , “Characteristics and Beach Safety Knowledge of Beachgoers on Unpatrolled Surf Beaches in Australia,” Natural Hazards and Earth System Sciences 22, no. 3 (2022): 909–926, 10.5194/nhess-22-909-2022.

[18] C. McKay , R. W. Brander , and J. Goff , “Putting Tourists in Harms Way – Coastal Tourist Parks and Hazardous Unpatrolled Surf Beaches in New South Wales, Australia,” Tourism Management 45, no. 1 (2014): 71–84, 10.1016/j.tourman.2014.03.007.

[19] A. Davies , L. Lockstone‐Binney , and K. Holmes , “Who Are the Future Volunteers in Rural Places? Understanding the Demographic and Background Characteristics of Non‐Retired Rural Volunteers, Why They Volunteer and Their Future Migration Intentions,” Journal of Rural Studies 60 (2018): 167–175, 10.1016/j.jrurstud.2018.04.003.

[20] B. R. Cook , P. Kamstra , N. Harrigan , et al., “Normative Learning Generates Behaviour Change: The Case of Drowning Prevention,” International Journal of Disaster Risk Reduction 114 (2024): 104942.

[21] O. P. Pastore , A. Blunden , A. E. Peden , J. C. Lawes , and R. W. Brander , “Evaluating Public Awareness and Perceptions of Emergency Response Beacons on Beaches of New South Wales, Australia,” Ocean and Coastal Management 262 (2025): 107553.

[22] S. L. Thomas , J. Wakerman , and J. S. Humphreys , “Ensuring Equity of Access to Primary Health Care in Rural and Remote Australia ‐ What Core Services Should Be Locally Available?,” International Journal for Equity in Health 14, no. 1 (2015): 111, 10.1186/s12939-015-0228-1. 26510998 PMC4625941

[23] A. T. Gregory , R. M. Armstrong , and M. B. Van Der Weyden , “Rural and Remote Health in Australia: How to Avert the Deepening Health Care Drought,” Medical Journal of Australia 185, no. 11–12 (2006): 654–660, 10.5694/j.1326-5377.2006.tb00744.x. 17181515

[24] A. H. Lee , L. B. Meuleners , Y. Zhao , M. Intrapanya , D. Palmer , and E. Mowatt , “Emergency Presentations to Northern Territory Public Hospitals: Demand and Access Analysis,” Australian Health Review 26, no. 2 (2003): 43–48, 10.1071/ah030043a. 15368835

[25] S. Sherker , A. Williamson , J. Hatfield , R. Brander , and A. Hayen , “Beachgoers' Beliefs and Behaviours in Relation to Beach Flags and Rip Currents,” Accident Analysis & Prevention 42, no. 6 (2010): 1785–1804, 10.1016/j.aap.2010.04.020. 20728630

[26] S. J. Pitman , K. Thompson , D. E. Hart , et al., “Beachgoers' Ability to Identify Rip Currents at a Beach In Situ,” Natural Hazards and Earth System Sciences 21, no. 1 (2021): 115–128, 10.5194/nhess-21-115-2021.

[27] A. D. Ménard , C. Houser , R. W. Brander , S. Trimble , and A. Scaman , “The Psychology of Beach Users: Importance of Confirmation Bias, Action, and Intention to Improving Rip Current Safety,” Natural Hazards 94, no. 2 (2018): 953–973, 10.1007/s11069-018-3424-7.

[28] S. Sherker , R. Brander , C. Finch , and J. Hatfield , “Why Australia Needs an Effective National Campaign to Reduce Coastal Drowning,” Journal of Science and Medicine in Sport 11, no. 2 (2008): 81–83, 10.1016/j.jsams.2006.08.007. 17118705

[29] W. Koon , R. W. Brander , G. Dusek , B. Castelle , and J. C. Lawes , “Relationships Between the Tide and Fatal Drowning at Surf Beaches in New South Wales, Australia: Implications for Coastal Safety Management and Practice,” Ocean and Coastal Management 238 (2023): 106584, 10.1016/j.ocecoaman.2023.106584.

[30] B. Castelle , T. Scott , R. Brander , et al., “Environmental Controls on Surf Zone Injuries on High‐Energy Beaches,” Natural Hazards and Earth Systems Science 19, no. 10 (2019): 2183–2205, 10.5194/nhess-19-2183-2019.

[31] F. Zhang and X. H. Wang , “Assessing Preferences of Beach Users for Certain Aspects of Weather and Ocean Conditions: Case Studies From Australia,” International Journal of Biometeorology 57, no. 3 (2013): 337–347, 10.1007/s00484-012-0556-4. 22661009

[32] J. C. Lawes , E. J. T. Rijksen , R. W. Brander , R. C. Franklin , and S. Daw , “Dying to Help: Fatal Bystander Rescues in Australian Coastal Environments,” PLoS One 15, no. 9 (2020): e0238317, 10.1371/journal.pone.0238317. 32936817 PMC7494089

[33] A. M. Venema , J. W. Groothoff , and J. J. L. M. Bierens , “The Role of Bystanders During Rescue and Resuscitation of Drowning Victims,” Resuscitation 81, no. 4 (2010): 434–439, 10.1016/j.resuscitation.2010.01.005. 20149515

[34] R. W. Brander , N. Warton , R. C. Franklin , W. S. Shaw , E. J. T. Rijksen , and S. Daw , “Characteristics of Aquatic Rescues Undertaken by Bystanders in Australia,” PLoS One 14, no. 2 (2019): e0212349, 10.1371/journal.pone.0212349. 30763388 PMC6375621

[35] World Health Organisation , Preventing Drowning: An Implementation Guide (World Health Organisation, 2017).

[36] C. Chung , L. Quan , E. Bennett , M. A. Kernic , and B. E. Ebel , “Informing Policy on Open Water Drowning Prevention: An Observational Survey of Life Jacket Use in Washington State,” Injury Prevention 20, no. 4 (2014): 238–243, 10.1136/injuryprev-2013-041005. 24513564

[37] D. A. Quistberg , E. Bennett , L. Quan , and B. E. Ebel , “Low Life Jacket Use Among Adult Recreational Boaters: A Qualitative Study of Risk Perception and Behavior Factors,” Accident; Analysis and Prevention 62 (2014): 276–284, 10.1016/j.aap.2013.10.015. 24211559 PMC3919505

[38] A. E. Peden , D. Demant , M. S. Hagger , and K. Hamilton , “Personal, Social, and Environmental Factors Associated With Lifejacket Wear in Adults and Children: A Systematic Literature Review,” PLoS One 13, no. 5 (2018): e0196421, 10.1371/journal.pone.0196421. 29718971 PMC5931488

[39] A. E. Peden , S. Daw , and J. C. Lawes , “Preliminary Evaluation of the Impact of Mandatory Life Jacket Laws at Declared High‐Risk Rock Platforms on Unintentional Rock Fishing Drowning Deaths,” Injury Prevention 28, no. 6 (2022): 560–563, 10.1136/ip-2022-044724. 36270790

[40] S. Willcox‐Pidgeon , A. E. Peden , R. C. Franklin , and J. Scarr , “Boating‐Related Drowning in Australia: Epidemiology, Risk Factors and the Regulatory Environment,” Journal of Safety Research 70 (2019): 117–125, 10.1016/j.jsr.2019.06.005. 31847986

