# Supplementary material for: Far From Help: Exploring the Influence of Regional and Remote Residence on Coastal Visitation and Participation, Risk Perception and Safety Knowledge and Practices
Source: Aust J Rural Health. 2025 Feb 24;33(1):e70018. doi: 10.1111/ajr.70018 (PMC11851053; doi:10.1111/ajr.70018)
Supplement: Supplementary file 1 — Table S1. Fishing and sailing/boating locations by remoteness. Table S2. Self‐reported risk perception to the coast and coastal activities by remoteness. Table S3. Self‐reported coastal safety knowledge and experiences by remoteness. Table S4. Self‐reported safety practices and equipment by coastal activity and remoteness. [file AJR-33-0-s001.docx]

**Appendix**

**Table S1. Fishing and sailing/boating locations by remoteness.**

|  | | **Remoteness Group** | | | | **Test statistic**  **(p-value)** |
| --- | --- | --- | --- | --- | --- | --- |
|  |  | **Major Cities** | | **Regional/Remote** | |  |
|  |  | **%** | **N** | **%** | **N** |  |
| **Fishing location** | Rocks | 14.9 | 164 | 17.8 | 144 | χ2 = 16.02  (**p=0.001**) |
|  | Beach | 36.0 | 397 | 41.4 | 335 |  |
|  | Jetty | 47.5 | 524 | 38.4 | 310 |  |
|  | Can't say | 1.7 | 19 | 2.3 | 19 |  |
| **Sailing/boating location** | Bay or harbour | 42.0 | 553 | 29.4 | 208 | χ2 = 47.12  (**p<0.001**) |
|  | Estuary, river mouth or mangrove | 42.0 | 541 | 29.3 | 238 |  |
|  | Offshore less than 2 (then 3 in 2022) nautical miles | 24.0 | 310 | 29.7 | 242 |  |
|  | Offshore more than 2 (then 3 in 2022) nautical miles | 18.0 | 232 | 20.5 | 167 |  |
|  | Can't say | 11.1 | 143 | 17.2 | 140 |  |

*Note:* *Significant chi-square analyses (p<0.05) are presented in bold.*

**Table S2. Self-reported risk perception to the coast and coastal activities by remoteness.**

|  | | | **Remoteness** | | | | **Test statistic (p-value)** |
| --- | --- | --- | --- | --- | --- | --- | --- |
|  |  |  | **Major Cities** | | **Regional/Remote** | |  |
|  |  |  | **%** | **N** | **%** | **N** |  |
| **Hazard perception** | The coast | Extremely hazardous | 8.1 | 761 | 10.7 | 517 | χ2 = 44.33  (**p<0.001**) |
|  |  | Very hazardous | 19.4 | 1812 | 20.7 | 1004 |  |
|  |  | Somewhat hazardous | 48.6 | 4552 | 47.1 | 2282 |  |
|  |  | Not very hazardous | 15.6 | 1461 | 13.9 | 673 |  |
|  |  | Not at all hazardous | 4.3 | 403 | 4.7 | 230 |  |
|  |  | Can't say | 4.0 | 372 | 3.0 | 143 |  |
|  | The beach | Extremely hazardous | 3.1 | 294 | 4.1 | 201 | χ2 = 40.74  (**p<0.001**) |
|  |  | Very hazardous | 8.6 | 809 | 10.7 | 518 |  |
|  |  | Somewhat hazardous | 42.9 | 4013 | 44.0 | 2131 |  |
|  |  | Not very hazardous | 32.5 | 3043 | 29.1 | 1413 |  |
|  |  | Not at all hazardous | 9.9 | 928 | 9.8 | 475 |  |
|  |  | Can't say | 2.9 | 274 | 2.3 | 111 |  |
|  | Swimming | Extremely hazardous | 2.7 | 253 | 4.1 | 198 | χ2 = 42.15  (**p<0.001**) |
|  |  | Very hazardous | 8.7 | 811 | 9.7 | 472 |  |
|  |  | Somewhat hazardous | 46.6 | 4367 | 48.2 | 2338 |  |
|  |  | Not very hazardous | 32.2 | 3019 | 29.4 | 1427 |  |
|  |  | Not at all hazardous | 6.7 | 624 | 6.3 | 306 |  |
|  |  | Can't say | 3.1 | 287 | 2.2 | 107 |  |
|  | Wading | Extremely hazardous | 1.4 | 128 | 1.7 | 81 | χ2 = 57.92  (**p<0.001**) |
|  |  | Very hazardous | 4.0 | 378 | 3.5 | 168 |  |
|  |  | Somewhat hazardous | 22.5 | 2109 | 20.6 | 998 |  |
|  |  | Not very hazardous | 43.5 | 4072 | 46.8 | 2267 |  |
|  |  | Not at all hazardous | 21.1 | 1974 | 22.8 | 1104 |  |
|  |  | Can't say | 7.5 | 700 | 4.8 | 231 |  |
|  | Surfing (including board surfing) | Extremely hazardous | 5.3 | 499 | 6.1 | 298 | χ2 = 10.50  (p=0.062) |
|  |  | Very hazardous | 18.8 | 1763 | 19.5 | 946 |  |
|  |  | Somewhat hazardous | 52.4 | 4902 | 52.7 | 2554 |  |
|  |  | Not very hazardous | 15.8 | 1481 | 14.9 | 720 |  |
|  |  | Not at all hazardous | 2.9 | 271 | 2.8 | 135 |  |
|  |  | Can't say | 4.8 | 445 | 4.0 | 195 |  |
|  | Other watercraft* | Extremely hazardous | 3.9 | 365 | 4.0 | 196 | χ2 =21.67  (**p<0.001**) |
|  |  | Very hazardous | 13.2 | 1240 | 13.6 | 661 |  |
|  |  | Somewhat hazardous | 50.9 | 4765 | 53.8 | 2607 |  |
|  |  | Not very hazardous | 22.9 | 2141 | 21.1 | 1025 |  |
|  |  | Not at all hazardous | 3.7 | 342 | 3.3 | 160 |  |
|  |  | Can't say | 5.4 | 509 | 4.2 | 201 |  |
|  | Rock fishing/fishing from rocky areas | Extremely hazardous | 21.9 | 2054 | 24.5 | 1186 | χ2 = 24.02  (**p<0.001**) |
|  |  | Very hazardous | 31.2 | 2918 | 31.8 | 1541 |  |
|  |  | Somewhat hazardous | 31.8 | 2979 | 30.0 | 1454 |  |
|  |  | Not very hazardous | 8.4 | 786 | 8.5 | 413 |  |
|  |  | Not at all hazardous | 2.5 | 238 | 2.1 | 104 |  |
|  |  | Can't say | 4.1 | 387 | 3.1 | 150 |  |
|  | Land-based fishing | Extremely hazardous | 1.9 | 182 | 1.9 | 90 | χ2 = 14.17  (**p=0.015**) |
|  |  | Very hazardous | 4.6 | 433 | 4.1 | 197 |  |
|  |  | Somewhat hazardous | 25.3 | 2365 | 25.1 | 1219 |  |
|  |  | Not very hazardous | 46.0 | 4302 | 47.9 | 2325 |  |
|  |  | Not at all hazardous | 17.6 | 1651 | 17.5 | 848 |  |
|  |  | Can't say | 4.6 | 428 | 3.5 | 169 |  |
|  | Boating (including boat-based fishing) | Extremely hazardous | 3.1 | 287 | 3.6 | 176 | χ2 = 19.73  (**p=0.001**) |
|  |  | Very hazardous | 10.5 | 981 | 10.6 | 516 |  |
|  |  | Somewhat hazardous | 48.3 | 4523 | 50.3 | 2440 |  |
|  |  | Not very hazardous | 28.4 | 2660 | 27.2 | 1318 |  |
|  |  | Not at all hazardous | 5.0 | 463 | 4.7 | 229 |  |
|  |  | Can't say | 4.8 | 447 | 3.5 | 170 |  |
|  | Snorkelling | Extremely hazardous | 2.9 | 271 | 4.4 | 216 | χ2 = 45.02  (**p<0.001**) |
|  |  | Very hazardous | 10.1 | 949 | 10.6 | 515 |  |
|  |  | Somewhat hazardous | 46.2 | 4322 | 47.8 | 2317 |  |
|  |  | Not very hazardous | 30.3 | 2834 | 28.0 | 1356 |  |
|  |  | Not at all hazardous | 4.6 | 435 | 4.8 | 234 |  |
|  |  | Can't say | 5.9 | 550 | 4.4 | 211 |  |
|  | Scuba diving | Extremely hazardous | 6.2 | 582 | 7.4 | 361 | χ2 = 22.96  (**p<0.001**) |
|  |  | Very hazardous | 21.9 | 2052 | 21.6 | 1046 |  |
|  |  | Somewhat hazardous | 47.0 | 4400 | 48.5 | 2351 |  |
|  |  | Not very hazardous | 15.8 | 1475 | 14.1 | 686 |  |
|  |  | Not at all hazardous | 3.0 | 277 | 3.3 | 162 |  |
|  |  | Can't say | 6.1 | 575 | 5.0 | 243 |  |
|  | Jet ski/Personal Watercraft | Extremely hazardous | 7.3 | 681 | 7.8 | 377 | χ2 = 7.40  (p=0.193) |
|  |  | Very hazardous | 22.6 | 2118 | 22.7 | 1101 |  |
|  |  | Somewhat hazardous | 47.1 | 4411 | 48.3 | 2341 |  |
|  |  | Not very hazardous | 14.7 | 1372 | 13.9 | 672 |  |
|  |  | Not at all hazardous | 2.7 | 255 | 2.5 | 124 |  |
|  |  | Can't say | 5.6 | 523 | 4.8 | 233 |  |
|  | Rip currents | Extremely hazardous | 43.7 | 4090 | 47.1 | 2284 | χ2 = 33.82  (**p<0.001**) |
|  |  | Very hazardous | 37.0 | 3467 | 35.2 | 1705 |  |
|  |  | Somewhat hazardous | 13.6 | 1275 | 13.9 | 672 |  |
|  |  | Not very hazardous | 1.9 | 182 | 1.3 | 64 |  |
|  |  | Not at all hazardous | 0.6 | 58 | 0.3 | 13 |  |
|  |  | Can't say | 3.1 | 289 | 2.3 | 111 |  |
|  | Waves | Extremely hazardous | 12.1 | 1130 | 11.4 | 553 | χ2 = 8.93  (p=0.112) |
|  |  | Very hazardous | 28.7 | 2684 | 28.2 | 1366 |  |
|  |  | Somewhat hazardous | 47.0 | 4396 | 48.6 | 2357 |  |
|  |  | Not very hazardous | 8.5 | 792 | 8.7 | 423 |  |
|  |  | Not at all hazardous | 1.0 | 92 | 0.9 | 43 |  |
|  |  | Can't say | 2.9 | 267 | 2.2 | 107 |  |
|  | Tropical marine stinger creatures, such as the box jellyfish or irukandji | Extremely hazardous | 36.8 | 3448 | 43.7 | 2117 | χ2 = 88.12  (**p<0.001**) |
|  |  | Very hazardous | 31.9 | 2987 | 30.9 | 1498 |  |
|  |  | Somewhat hazardous | 23.2 | 2170 | 19.9 | 967 |  |
|  |  | Not very hazardous | 3.5 | 329 | 2.3 | 112 |  |
|  |  | Not at all hazardous | 0.8 | 78 | 0.3 | 16 |  |
|  |  | Can't say | 3.7 | 350 | 2.9 | 140 |  |
|  | Other marine stinger creatures such as bluebottles | Extremely hazardous | 24.7 | 2315 | 30.1 | 1459 | χ2 = 70.30  (**p<0.001**) |
|  |  | Very hazardous | 34.8 | 3260 | 34.6 | 1677 |  |
|  |  | Somewhat hazardous | 31.0 | 2897 | 28.4 | 1376 |  |
|  |  | Not very hazardous | 4.9 | 456 | 3.8 | 183 |  |
|  |  | Not at all hazardous | 0.9 | 88 | 0.4 | 20 |  |
|  |  | Can't say | 3.7 | 345 | 2.8 | 133 |  |
|  | Sharks | Extremely hazardous | 39.7 | 3720 | 38.0 | 1841 | χ2 = 22.53  (**p<0.001**) |
|  |  | Very hazardous | 24.7 | 2314 | 24.9 | 1207 |  |
|  |  | Somewhat hazardous | 23.6 | 2205 | 26.1 | 1266 |  |
|  |  | Not very hazardous | 8.0 | 748 | 8.1 | 395 |  |
|  |  | Not at all hazardous | 1.1 | 107 | 0.8 | 41 |  |
|  |  | Can't say | 2.9 | 267 | 2.0 | 98 |  |
|  | Crocodiles | Extremely hazardous | 41.9 | 3922 | 46.6 | 2258 | χ2 = 68.05  (**p<0.001**) |
|  |  | Very hazardous | 22.1 | 2070 | 22.3 | 1082 |  |
|  |  | Somewhat hazardous | 17.8 | 1665 | 17.8 | 865 |  |
|  |  | Not very hazardous | 9.2 | 863 | 6.6 | 319 |  |
|  |  | Not at all hazardous | 3.9 | 369 | 2.4 | 118 |  |
|  |  | Can't say | 5.0 | 472 | 4.3 | 207 |  |
|  | Sun exposure | Extremely hazardous | 28.7 | 2685 | 30.1 | 1462 | χ2 = 21.92  (**p<0.001**) |
|  |  | Very hazardous | 37.8 | 3538 | 39.2 | 1899 |  |
|  |  | Somewhat hazardous | 26.1 | 2442 | 25.1 | 1219 |  |
|  |  | Not very hazardous | 4.1 | 387 | 3.0 | 145 |  |
|  |  | Not at all hazardous | 0.7 | 68 | 0.6 | 29 |  |
|  |  | Can't say | 2.6 | 240 | 2.0 | 95 |  |
|  | Rocks/Rocky platform | Extremely hazardous | 23.2 | 1932 | 24.2 | 1023 | χ2 =12.62  (**p=0.027**) |
|  |  | Very hazardous | 39.4 | 3286 | 38.2 | 1615 |  |
|  |  | Somewhat hazardous | 30.3 | 2532 | 31.5 | 1330 |  |
|  |  | Not very hazardous | 3.4 | 282 | 3.3 | 139 |  |
|  |  | Not at all hazardous | 0.6 | 49 | 0.4 | 16 |  |
|  |  | Can't say | 3.2 | 265 | 2.4 | 99 |  |
| **Level of concern** | The water quality | Extremely concerned | 9.6 | 607 | 8.7 | 257 | χ2 = 25.60  (**p<0.001**) |
|  |  | Very concerned | 21.2 | 1339 | 18.9 | 562 |  |
|  |  | Somewhat concerned | 34.4 | 2169 | 34.0 | 1010 |  |
|  |  | Not very concerned | 23.5 | 1486 | 25.1 | 745 |  |
|  |  | Not at all concerned | 7.4 | 469 | 9.9 | 295 |  |
|  |  | Can't say | 3.8 | 239 | 3.4 | 100 |  |
|  | Being hit in the water by a board or a watercraft | Extremely concerned | 10.0 | 634 | 8.1 | 240 | χ2 = 94.10  (**p<0.001**) |
|  |  | Very concerned | 16.1 | 1016 | 12.4 | 370 |  |
|  |  | Somewhat concerned | 30.1 | 1899 | 26.2 | 777 |  |
|  |  | Not very concerned | 30.9 | 1949 | 36.3 | 1079 |  |
|  |  | Not at all concerned | 8.7 | 550 | 13.1 | 389 |  |
|  |  | Can't say | 4.1 | 261 | 3.9 | 115 |  |
|  | Being stung by a jellyfish (i.e. bluebottles) | Extremely concerned | 13.9 | 877 | 14.5 | 430 | χ2 = 29.59  (**p<0.001**) |
|  |  | Very concerned | 20.9 | 1321 | 19.5 | 578 |  |
|  |  | Somewhat concerned | 36.1 | 2278 | 34.2 | 1015 |  |
|  |  | Not very concerned | 21.5 | 1358 | 22.8 | 676 |  |
|  |  | Not at all concerned | 4.1 | 262 | 6.4 | 189 |  |
|  |  | Can't say | 3.4 | 214 | 2.7 | 80 |  |
|  | Being caught unintentionally in a rip | Extremely concerned | 19.9 | 1256 | 19.1 | 566 | χ2 = 66.02  (**p<0.001**) |
|  |  | Very concerned | 26.2 | 1653 | 22.0 | 652 |  |
|  |  | Somewhat concerned | 32.5 | 2047 | 33.0 | 979 |  |
|  |  | Not very concerned | 14.4 | 911 | 16.5 | 488 |  |
|  |  | Not at all concerned | 3.4 | 216 | 6.5 | 193 |  |
|  |  | Can't say | 3.6 | 226 | 3.0 | 90 |  |
| **Perceived experience to take risk** | Swimming or wading | No | 48.2 | 2201 | 45.0 | 949 | χ2 = 6.03  (**p=0.014**) |
|  |  | Yes | 51.8 | 2365 | 55.0 | 1161 |  |
|  | Surfing (including surfboards and body boards) | No | 48.2 | 319 | 46.1 | 117 | χ2 = 0.33  (p=0.564) |
|  |  | Yes | 51.8 | 343 | 53.9 | 137 |  |
|  | Other watercraft* | No | 52.3 | 412 | 52.0 | 205 | χ2 = 0.01  (p=0.917) |
|  |  | Yes | 47.7 | 375 | 48.0 | 189 |  |
|  | Rock fishing | No | 52.1 | 184 | 41.5 | 105 | χ2 = 6.67  (**p=0.010**) |
|  |  | Yes | 47.9 | 169 | 58.5 | 148 |  |
|  | Land-based fishing | No | 48.5 | 470 | 41.9 | 305 | χ2 = 7.08  (**p=0.008**) |
|  |  | Yes | 51.5 | 500 | 58.1 | 422 |  |
|  | Boating (including boat-based fishing) | No | 58.8 | 762 | 57.4 | 470 | χ2 = 0.42  (p=0.522) |
|  |  | Yes | 41.2 | 534 | 42.6 | 349 |  |
|  | Snorkelling | No | 48.2 | 491 | 45.6 | 206 | χ2 = 0.82  (p=0.366) |
|  |  | Yes | 51.8 | 527 | 54.4 | 245 |  |
|  | Scuba diving | No | 46.9 | 108 | 44.9 | 37 | χ2 = 0.12  (p=0.733) |
|  |  | Yes | 53.1 | 123 | 55.1 | 46 |  |
|  | Jet ski/Personal Watercraft | No | 52.4 | 201 | 46.7 | 80 | χ2 = 1.46  (p=0.226) |
|  |  | Yes | 47.6 | 183 | 53.3 | 91 |  |

*Notes:*

- *Significant chi-square analyses (p<0.05) are presented in bold.*
- **Other watercraft includes paddle craft, stand-up-paddle, paddle boarding, kite surfing and wind surfing.*

**Table S3. Self-reported coastal safety knowledge and experiences by remoteness.**

|  | | | **Remoteness** | | | | **Test statistic**  **(p-value)** |
| --- | --- | --- | --- | --- | --- | --- | --- |
|  |  |  | **Major Cities** | | **Regional/ Remote** | |  |
|  |  |  | **%** | **N** | **%** | **N** |  |
| **Been rescued** | Swimming or wading | Yes | 6.4 | 328 | 5.1 | 125 | χ2 = 4.79  (**p=0.029**) |
|  |  | No | 93.6 | 4811 | 94.9 | 2322 |  |
|  | Surfing (including surfboards and body boards) | Yes | 7.2 | 54 | 3.6 | 11 | χ2 = 4.89  (**p=0.027**) |
|  |  | No | 92.8 | 691 | 96.4 | 293 |  |
|  | Other watercraft* | Yes | 6.7 | 58 | 3.7 | 16 | χ2 = 4.65  (**p=0.031**) |
|  |  | No | 93.3 | 804 | 96.3 | 410 |  |
|  | Rock fishing/fishing from rocky areas | Yes | 7.1 | 29 | 2.1 | 6 | χ2 = 9.80  (**p=0.002**) |
|  |  | No | 92.9 | 386 | 97.9 | 292 |  |
|  | Land-based fishing | Yes | 2.6 | 28 | 0.5 | 4 | χ2 = 12.71  (**p<0.001**) |
|  |  | No | 97.4 | 1079 | 99.5 | 848 |  |
|  | Boating | Yes | 4.7 | 67 | 4.7 | 44 | χ2 = 0.00  (p=0.986) |
|  |  | No | 95.3 | 1357 | 95.3 | 888 |  |
|  | Snorkelling | Yes | 2.3 | 26 | 0.4 | 2 | χ2 = 7.57  (**p=0.006**) |
|  |  | No | 97.7 | 1112 | 99.6 | 509 |  |
|  | Scuba diving | Yes | 6.2 | 16 | 8.9 | 9 | χ2 = 0.80  (p=0.372) |
|  |  | No | 93.8 | 238 | 91.1 | 91 |  |
|  | Jet ski/Personal Watercraft | Yes | 7.0 | 28 | 5.8 | 11 | χ2 = 0.24  (p=0.621) |
|  |  | No | 93.0 | 378 | 94.2 | 178 |  |
| **Rescued someone** | Swimming or wading | Yes | 14.7 | 756 | 17.0 | 415 | χ2 = 6.44  (**p=0.011**) |
|  |  | No | 85.3 | 4382 | 83.0 | 2031 |  |
|  | Surfing (including surfboards and body boards) | Yes | 16.8 | 125 | 16.1 | 49 | χ2 = 0.06  (p=0.811) |
|  |  | No | 83.2 | 620 | 83.9 | 254 |  |
|  | Other watercraft* | Yes | 9.9 | 85 | 9.6 | 41 | χ2 = 0.02  (p=0.893) |
|  |  | No | 90.1 | 777 | 90.4 | 385 |  |
|  | Rock fishing/fishing from rocky areas | Yes | 11.4 | 47 | 6.5 | 19 | χ2 = 5.06  (**p=0.025**) |
|  |  | No | 88.6 | 368 | 93.5 | 279 |  |
|  | Land-based fishing | Yes | 3.6 | 40 | 2.2 | 19 | χ2 =3.16  (p=0.076) |
|  |  | No | 96.4 | 1068 | 97.8 | 834 |  |
|  | Boating (including boat-based fishing) | Yes | 10.3 | 146 | 13.6 | 127 | χ2 = 6.23  (**p=0.013**) |
|  |  | No | 89.7 | 1277 | 86.4 | 805 |  |
|  | Snorkelling | Yes | 5.2 | 60 | 6.9 | 35 | χ2 = 1.62  (p=0.204) |
|  |  | No | 94.8 | 1078 | 93.1 | 476 |  |
|  | Scuba diving | Yes | 16.7 | 42 | 29.8 | 30 | χ2 = 7.93  (**p=0.005**) |
|  |  | No | 83.3 | 211 | 70.2 | 70 |  |
|  | Jet ski/Personal Watercraft | Yes | 12.8 | 52 | 7.5 | 14 | χ2 = 3.70  (p=0.054) |
|  |  | No | 87.2 | 355 | 92.5 | 174 |  |
| **Last time being rescued** | Location | Beach (less than 500 m from shore) | 46.6 | 191 | 47.3 | 77 | χ2 = 11.25  (**p=0.047**) |
|  |  | Rocky coast (eg shore platform, cliffs, rocky headland, reef) | 15.1 | 62 | 8.1 | 13 |  |
|  |  | Open ocean (more than 500m from shore) | 20.7 | 84 | 22.9 | 37 |  |
|  |  | Jetty / pier / marina | 2.9 | 12 | 0.7 | 1 |  |
|  |  | Somewhere else | 10.6 | 43 | 16.4 | 27 |  |
|  |  | Can't say, can't remember | 4.1 | 17 | 4.7 | 8 |  |
|  | Time | 12am - 4am | 3.1 | 13 | 5.8 | 9 | χ2 = 10.12  (p=0.120) |
|  |  | 4am - 8am | 6.1 | 25 | 6.1 | 10 |  |
|  |  | 8am - 12pm | 24.7 | 101 | 15.2 | 25 |  |
|  |  | 12pm - 4pm | 45.3 | 185 | 52.5 | 85 |  |
|  |  | 4pm - 8pm | 8.2 | 34 | 11.1 | 18 |  |
|  |  | 8pm - 12am | 1.6 | 7 | 0.6 | 1 |  |
|  |  | Can't say, can't remember | 11.0 | 45 | 8.8 | 14 |  |
|  | Lifeguards or lifesavers on duty? | Yes | 38.9 | 159 | 33.3 | 54 | χ2 = 14.21  (**p=0.048**) |
|  |  | No, but there were lifeguards patrolling less than 1km away | 7.5 | 31 | 4.1 | 7 |  |
|  |  | No, but there were lifeguards or lifesavers patrolling between 1km and 5km away | 5.0 | 21 | 2.7 | 4 |  |
|  |  | No, but there were lifeguards or lifesavers patrolling more than 5km away | 2.7 | 11 | 1.5 | 2 |  |
|  |  | No lifeguards / it was outside patrol hours | 6.4 | 26 | 11.8 | 19 |  |
|  |  | No lifeguards at this patrolled beach as it was outside patrol hours | 7.3 | 30 | 7.5 | 12 |  |
|  |  | No lifeguards as it was at an unpatrolled beach | 19.2 | 79 | 27.8 | 45 |  |
|  |  | Can't say, can't remember | 13.0 | 53 | 11.4 | 18 |  |
|  | Who performed the rescue? | Someone I knew | 32.9 | 134 | 39.5 | 64 | χ2 = 5.18  (p=0.159) |
|  |  | Lifeguard | 35.1 | 143 | 25.5 | 41 |  |
|  |  | Someone else, a stranger | 26.5 | 108 | 29.3 | 47 |  |
|  |  | Can't say, can't remember | 5.5 | 23 | 5.8 | 9 |  |
|  | Floatation device used? | A board | 19.9 | 81 | 15.5 | 25 | χ2 = 17.63  (**p=0.007**) |
|  |  | A tube | 11.8 | 48 | 8.9 | 14 |  |
|  |  | An angel ring / a life buoy | 10.4 | 43 | 4.0 | 7 |  |
|  |  | A lifejacket | 8.8 | 36 | 5.8 | 9 |  |
|  |  | Another flotation device or a watercraft | 13.6 | 55 | 15.1 | 25 |  |
|  |  | None | 27.8 | 114 | 43.2 | 70 |  |
|  |  | Can't say, can't remember | 7.7 | 31 | 7.5 | 12 |  |
| **Last time performing a rescue** | Location | Beach (less than 500 m from shore) | 55.4 | 430 | 50.4 | 210 | χ2 = 8.29  (p=0.141) |
|  |  | Rocky coast (eg shore platform, cliffs, rocky headland, reef) | 8.1 | 63 | 7.9 | 33 |  |
|  |  | Open ocean (more than 500m from shore) | 13.2 | 102 | 15.8 | 66 |  |
|  |  | Jetty / pier / marina | 4.4 | 34 | 3.7 | 15 |  |
|  |  | Somewhere else | 15.3 | 119 | 20.0 | 83 |  |
|  |  | Can't say, can't remember | 3.6 | 28 | 2.1 | 9 |  |
|  | Time | 12am - 4am | 1.6 | 13 | 4.2 | 17 | χ2 = 18.54  (**p=0.005**) |
|  |  | 4am - 8am | 4.1 | 32 | 3.3 | 14 |  |
|  |  | 8am - 12pm | 26.3 | 204 | 21.3 | 89 |  |
|  |  | 12pm - 4pm | 47.8 | 370 | 47.1 | 196 |  |
|  |  | 4pm - 8pm | 9.9 | 77 | 8.0 | 33 |  |
|  |  | 8pm - 12am | 1.0 | 8 | 1.8 | 8 |  |
|  |  | Can't say, can't remember | 9.3 | 72 | 14.3 | 60 |  |
|  | Lifeguards or lifesavers on duty? | Yes | 30.1 | 233 | 20.8 | 86 | χ2 = 33.41  (**p<0.001**) |
|  |  | No, but there were lifeguards patrolling less than 1km away | 6.1 | 47 | 4.6 | 19 |  |
|  |  | No, but there were lifeguards or lifesavers patrolling between 1km and 5km away | 4.2 | 33 | 2.7 | 11 |  |
|  |  | No, but there were lifeguards or lifesavers patrolling more than 5km away | 3.7 | 28 | 2.0 | 8 |  |
|  |  | No lifeguards / it was outside patrol hours | 13.1 | 101 | 18.4 | 76 |  |
|  |  | No lifeguards at this patrolled beach as it was outside patrol hours | 9.1 | 70 | 6.2 | 26 |  |
|  |  | No lifeguards as it was at an unpatrolled beach | 22.9 | 177 | 33.3 | 138 |  |
|  |  | Can't say, can't remember | 11.0 | 85 | 12.2 | 51 |  |
|  | Who did you rescue? | Someone I knew | 43.0 | 333 | 39.9 | 166 | χ2 = 10.30  (**p=0.006**) |
|  |  | Someone else, a stranger | 51.3 | 398 | 57.9 | 241 |  |
|  |  | Can't say | 5.7 | 44 | 2.2 | 9 |  |
|  | Floatation device used? | A board | 16.8 | 130 | 11.1 | 46 | χ2 = 14.61  (**p=0.024**) |
|  |  | A tube | 7.3 | 56 | 6.4 | 27 |  |
|  |  | An angel ring / a life buoy | 6.0 | 47 | 3.3 | 14 |  |
|  |  | A lifejacket | 7.1 | 55 | 7.5 | 31 |  |
|  |  | Another flotation device or a watercraft | 8.3 | 65 | 9.9 | 41 |  |
|  |  | None | 50.8 | 394 | 58.9 | 245 |  |
|  |  | Can't say, can't remember | 3.7 | 29 | 2.9 | 12 |  |
|  |  | Selected | 1.7 | 45 | 2.1 | 27 |  |
| **Rip currents** | Caught in a rip current? | Yes | 20.2 | 1892 | 20.0 | 971 | χ2 = 0.07  (p=0.792) |
|  |  | No | 79.8 | 7469 | 80.0 | 3878 |  |
|  | Lifeguards or lifesavers present when caught in rip current? | Yes | 42.2 | 799 | 32.3 | 314 | χ2 = 56.22  (**p<0.001**) |
|  |  | No, but there were lifeguards patrolling less than 1km away | 7.0 | 132 | 5.8 | 56 |  |
|  |  | No, but there were lifeguards or lifesavers patrolling between 1km and 5km away | 1.5 | 28 | 2.2 | 22 |  |
|  |  | No, but there were lifeguards or lifesavers patrolling more than 5km away | 2.7 | 51 | 2.6 | 25 |  |
|  |  | No lifeguards / it was outside patrol hours | 12.9 | 244 | 21.4 | 207 |  |
|  |  | No lifeguards at this patrolled beach as it was outside patrol hours | 6.2 | 117 | 4.8 | 47 |  |
|  |  | No lifeguards as it was at an unpatrolled beach | 14.6 | 276 | 18.2 | 177 |  |
|  |  | Can't say, can't remember | 13.0 | 247 | 12.7 | 124 |  |
|  | How did you get out of the rip current? | I swam and managed to get out by myself | 61.3 | 1160 | 61.6 | 598 | χ2 = 14.42  (**p=0.025**) |
|  |  | A surfer helped me / rescued me | 6.2 | 118 | 3.7 | 36 |  |
|  |  | A lifesaver or a lifeguard helped me / rescued me | 6.3 | 119 | 4.8 | 47 |  |
|  |  | Someone else helped me | 11.8 | 224 | 14.7 | 143 |  |
|  |  | I floated with the current and it returned me to shore | 10.4 | 197 | 10.9 | 106 |  |
|  |  | Other (please specify) | 2.4 | 45 | 2.5 | 24 |  |
|  |  | Can't say | 1.6 | 30 | 1.7 | 17 |  |
| **Where would you turn to for coastal safety information?** | Bureau of Meteorology | Not Selected | 55.3 | 4938 | 47.3 | 2169 | χ2 = 78.38  (**p<0.001**) |
|  |  | Selected | 44.7 | 3987 | 52.7 | 2417 |  |
|  | Weatherzone | Not Selected | 76.1 | 6791 | 71.2 | 3266 | χ2 = 37.80  (**p<0.001**) |
|  |  | Selected | 23.9 | 2134 | 28.8 | 1320 |  |
|  | State government maritime agency | Not Selected | 81.1 | 7240 | 78.4 | 3595 | χ2 = 14.21  (**p<0.001**) |
|  |  | Selected | 18.9 | 1685 | 21.6 | 991 |  |
|  | Coast guards | Not Selected | 59.7 | 5326 | 59.8 | 2743 | χ2 = 0.02  (p=0.877) |
|  |  | Selected | 40.3 | 3599 | 40.2 | 1843 |  |
|  | Lifeguards | Not Selected | 42.4 | 3782 | 46.7 | 2143 | χ2 = 23.32  (**p<0.001**) |
|  |  | Selected | 57.6 | 5143 | 53.3 | 2443 |  |
|  | Surf Life Saving or lifesavers/surf lifesavers | Not Selected | 48.2 | 4297 | 49.0 | 2249 | χ2 = 0.97  (p=0.324) |
|  |  | Selected | 51.8 | 4628 | 51.0 | 2337 |  |
|  | Marine rescue organisation | Not Selected | 83.4 | 7445 | 78.7 | 3611 | χ2 = 44.58  (**p<0.001**) |
|  |  | Selected | 16.6 | 1480 | 21.3 | 975 |  |
|  | Swimming/surfing/fishing/boating/snorkelling/diving club or organisation/surf school/scuba diving company | Not Selected | 82.9 | 7399 | 82.6 | 3790 | χ2 = 0.14  (p=0.705) |
|  |  | Selected | 17.1 | 1526 | 17.4 | 796 |  |
|  | Beachsafe | Not Selected | 81.9 | 7306 | 85.3 | 3913 | χ2 = 25.95  (**p<0.001**) |
|  |  | Selected | 18.1 | 1620 | 14.7 | 673 |  |
|  | Coastalwatch | Not Selected | 69.4 | 6194 | 66.2 | 3038 | χ2 = 13.94  (**p<0.001**) |
|  |  | Selected | 30.6 | 2731 | 33.8 | 1548 |  |
|  | Swellnet | Not Selected | 96.3 | 8595 | 96.5 | 4426 | χ2 = 0.38  (p=0.539) |
|  |  | Selected | 3.7 | 330 | 3.5 | 160 |  |
|  | Local council | Not Selected | 81.1 | 7235 | 82.4 | 3779 | χ2 = 3.60  (p=0.058) |
|  |  | Selected | 18.9 | 1690 | 17.6 | 807 |  |
|  | Other | Not Selected | 99.2 | 8851 | 99.0 | 4538 | χ2 = 1.60  (p=0.206) |
|  |  | Selected | 0.8 | 74 | 1.0 | 48 |  |
|  | Can't say | Not Selected | 90.7 | 8095 | 92.3 | 4233 | χ2 = 9.74  (**p=0.002**) |
|  |  | Selected | 9.3 | 830 | 7.7 | 353 |  |
| **Where do you receive coastal safety information from?** | Online | Not Selected | 44.3 | 3953 | 46.4 | 2128 | χ2 = 5.45  (**p=0.020**) |
|  |  | Selected | 55.7 | 4972 | 53.6 | 2458 |  |
|  | TV | Not Selected | 84.8 | 7570 | 86.7 | 3975 | χ2 = 8.42  (**p=0.004**) |
|  |  | Selected | 15.2 | 1355 | 13.3 | 611 |  |
|  | Radio | Not Selected | 89.6 | 7995 | 88.3 | 4048 | χ2 = 5.38  (**p=0.020**) |
|  |  | Selected | 10.4 | 930 | 11.7 | 538 |  |
|  | Newspaper | Not Selected | 94.5 | 8430 | 94.1 | 4315 | χ2 = 0.75  (p=0.388) |
|  |  | Selected | 5.5 | 495 | 5.9 | 271 |  |
|  | Magazine | Not Selected | 98.0 | 8748 | 98.9 | 4535 | χ2 = 13.86  (**p<0.001**) |
|  |  | Selected | 2.0 | 177 | 1.1 | 51 |  |
|  | Regular email newsletter | Not Selected | 97.7 | 8721 | 98.6 | 4522 | χ2 = 12.35  (**p<0.001**) |
|  |  | Selected | 2.3 | 204 | 1.4 | 64 |  |
|  | Using an app on smartphone or tablet | Not Selected | 80.2 | 7158 | 78.0 | 3578 | χ2 = 8.84  (**p=0.003**) |
|  |  | Selected | 19.8 | 1767 | 22.0 | 1008 |  |
|  | Other | Not Selected | 97.4 | 8697 | 96.5 | 4428 | χ2 = 8.66  (**p=0.003**) |
|  |  | Selected | 2.6 | 228 | 3.5 | 158 |  |
|  | None of these/can't say | Not Selected | 73.1 | 6528 | 71.4 | 3275 | χ2 = 4.51  (**p=0.034**) |
|  |  | Selected | 26.9 | 2398 | 28.6 | 1311 |  |
| **Have you seen, read or heard any advertising about rip currents from Surf Life Saving Australia in the last 3 months?** | | Yes | 14.8 | 1388 | 17.2 | 835 | χ2 = 13.90  (**p<0.001**) |
|  |  | No | 85.2 | 7973 | 82.8 | 4013 |  |
| **Rip current safety poster** | Have you seen this poster before today?* | Yes | 12.7 | 1189 | 11.8 | 572 | χ2 = 2.41  (p=0.120) |
|  |  | No | 87.3 | 8172 | 88.2 | 4277 |  |
|  | The message on this poster is clear and easy to understand | Yes | 86.0 | 8051 | 89.2 | 4327 | χ2 = 36.80  (**p<0.001**) |
|  |  | No | 9.5 | 888 | 6.6 | 318 |  |
|  |  | Can't say | 4.5 | 423 | 4.2 | 204 |  |
|  | You feel better informed on how to get out of a rip current | Yes | 84.2 | 7884 | 85.6 | 4148 | χ2 = 5.53  (p=0.063) |
|  |  | No | 9.5 | 892 | 8.4 | 406 |  |
|  |  | Can't say | 6.3 | 586 | 6.1 | 294 |  |
|  | You have just learned that different options exist to get out of a rip | Yes | 81.5 | 7634 | 82.2 | 3985 | χ2 = 1.11  (p=0.573) |
|  |  | No | 12.1 | 1129 | 11.5 | 556 |  |
|  |  | Can't say | 6.4 | 598 | 6.3 | 308 |  |
| **What option would you personally use to get out of a rip?** | Raise an arm and call out to seek help | Not Selected | 35.2 | 3299 | 35.1 | 1702 | χ2 = 0.03  (p=0.867) |
|  |  | Selected | 64.8 | 6062 | 64.9 | 3147 |  |
|  | Try floating with the current, as it may bring me back to shore | Not Selected | 65.0 | 6085 | 64.7 | 3137 | χ2 = 0.14  (p=0.714) |
|  |  | Selected | 35.0 | 3276 | 35.3 | 1712 |  |
|  | Swim parallel to the shore or towards breaking waves and use them to help me in | Not Selected | 30.5 | 2858 | 26.5 | 1283 | χ2 = 25.65  (**p<0.001**) |
|  |  | Selected | 69.5 | 6503 | 73.5 | 3566 |  |
|  | Would do something else | Not Selected | 98.9 | 9255 | 98.3 | 4766 | χ2 = 7.65  (**p=0.006**) |
|  |  | Selected | 1.1 | 106 | 1.7 | 82 |  |
|  | None of these/can't say | Not Selected | 93.3 | 8733 | 94.7 | 4593 | χ2 = 11.19  (**p<0.001**) |
|  |  | Selected | 6.7 | 628 | 5.3 | 256 |  |
|  |  | No | 3.7 | 345 | 2.2 | 106 |  |
|  |  | Can't say | 9.8 | 921 | 7.8 | 379 |  |

*Notes:*

- *Significant chi-square analyses (p<0.05) are presented in bold.*
- **Other watercraft includes paddle craft, stand-up-paddle, paddle boarding, kite surfing and wind surfing.*

**Table S4. Self-reported safety practices and equipment by coastal activity and remoteness.**

|  | | | **Remoteness** | | | | **Test statistic (p-value)** |
| --- | --- | --- | --- | --- | --- | --- | --- |
|  |  |  | **MC** | | **RR** | |  |
|  |  |  | **%** | **N** | **%** | **N** |  |
| **Lifejackets at home** | | Yes | 14.0 | 1312 | 21.4 | 1039 | χ2 = 127.08  (**p<0.001**) |
|  |  | No | 86.0 | 8049 | 78.6 | 3810 |  |
| **Wearing lifejackets during certain activities** | Swimming | Not Selected | 83.9 | 1101 | 90.8 | 943 | χ2 = 23.91  (**p<0.001**) |
|  |  | Selected | 16.1 | 211 | 9.2 | 96 |  |
|  | Wading | Not Selected | 96.1 | 1262 | 97.8 | 1016 | χ2 = 5.31  (**p=0.021**) |
|  |  | Selected | 3.9 | 51 | 2.2 | 23 |  |
|  | Surfing | Not Selected | 90.4 | 1187 | 95.7 | 994 | χ2 = 24.80  (**p<0.001**) |
|  |  | Selected | 9.6 | 126 | 4.3 | 44 |  |
|  | Body boarding | Not Selected | 93.9 | 1232 | 97.2 | 1009 | χ2 = 14.30  (**p<0.001**) |
|  |  | Selected | 6.1 | 80 | 2.8 | 29 |  |
|  | Paddle craft | Not Selected | 82.7 | 1085 | 88.2 | 916 | χ2 = 13.66  (**p<0.001**) |
|  |  | Selected | 17.3 | 227 | 11.8 | 123 |  |
|  | Stand-up-paddle/paddle boarding | Not Selected | 87.2 | 1145 | 92.6 | 962 | χ2 = 17.63  (**p<0.001**) |
|  |  | Selected | 12.8 | 167 | 7.4 | 77 |  |
|  | Kite surfing | Not Selected | 93.0 | 1220 | 95.7 | 994 | χ2 = 8.18  (**p=0.004**) |
|  |  | Selected | 7.0 | 92 | 4.3 | 44 |  |
|  | Wind surfing | Not Selected | 91.0 | 1194 | 95.5 | 992 | χ2 = 17.76  (**p<0.001**) |
|  |  | Selected | 9.0 | 118 | 4.5 | 47 |  |
|  | Rock fishing/fishing from rocky areas | Not Selected | 90.4 | 1186 | 93.6 | 972 | χ2 = 7.97  (**p=0.005**) |
|  |  | Selected | 9.6 | 127 | 6.4 | 67 |  |
|  | Land-based fishing | Not Selected | 96.2 | 1263 | 98.4 | 1022 | χ2 = 9.36  (**p=0.002**) |
|  |  | Selected | 3.8 | 49 | 1.6 | 17 |  |
|  | Boat-based fishing | Not Selected | 65.5 | 859 | 52.5 | 545 | χ2 = 40.52  (**p<0.001**) |
|  |  | Selected | 34.5 | 453 | 47.5 | 493 |  |
|  | Boating (power boat) | Not Selected | 52.8 | 693 | 49.0 | 508 | χ2 = 3.49  (p=0.062) |
|  |  | Selected | 47.2 | 619 | 51.0 | 530 |  |
|  | Sailing | Not Selected | 80.5 | 1057 | 83.7 | 869 | χ2 = 4.05  (**p=0.044**) |
|  |  | Selected | 19.5 | 256 | 16.3 | 169 |  |
|  | Water skiing or wakeboarding | Not Selected | 80.0 | 1050 | 81.3 | 845 | χ2 = 0.68  (p=0.408) |
|  |  | Selected | 20.0 | 263 | 18.7 | 194 |  |
|  | Snorkelling | Not Selected | 94.0 | 1234 | 97.2 | 1009 | χ2 = 13.24  (**p<0.001**) |
|  |  | Selected | 6.0 | 78 | 2.8 | 29 |  |
|  | Scuba diving | Not Selected | 95.5 | 1253 | 98.6 | 1024 | χ2 = 19.73  (**p<0.001**) |
|  |  | Selected | 4.5 | 60 | 1.4 | 14 |  |
|  | Jet ski/Personal Watercraft | Not Selected | 74.0 | 971 | 80.7 | 838 | χ2 = 14.78  (**p<0.001**) |
|  |  | Selected | 26.0 | 341 | 19.3 | 200 |  |
|  | Kayaking- canoeing | Not Selected | 68.6 | 901 | 65.5 | 680 | χ2 = 2.65  (p=0.103) |
|  |  | Selected | 31.4 | 412 | 34.5 | 359 |  |
|  | Other (please specify) | Not Selected | 98.4 | 1291 | 97.3 | 1010 | χ2 = 3.41  (p=0.065) |
|  |  | Selected | 1.6 | 21 | 2.7 | 28 |  |
|  | Can't say | Not Selected | 95.3 | 1251 | 94.7 | 983 | χ2 = 0.52  (p=0.471) |
|  |  | Selected | 4.7 | 61 | 5.3 | 55 |  |
|  |  | No \ do not have | 68.8 | 83 | 77.2 | 108 |  |
| **Swimming or wading safety practices** | Swim or wade at a patrolled beach during patrol times | Always | 37.2 | 1772 | 29.4 | 647 | χ2 = 240.14  (**p<0.001**) |
|  |  | Most of the time | 32.6 | 1550 | 24.0 | 528 |  |
|  |  | Sometimes | 23.9 | 1140 | 30.6 | 672 |  |
|  |  | Never | 3.6 | 173 | 9.7 | 214 |  |
|  |  | Can't say | 2.6 | 126 | 6.2 | 137 |  |
|  | Swim or wade between the red and yellow flags when you are on a patrolled beach | Always | 53.1 | 2530 | 53.0 | 1164 | χ2 = 76.15  (**p<0.001**) |
|  |  | Most of the time | 27.1 | 1291 | 22.6 | 496 |  |
|  |  | Sometimes | 14.5 | 691 | 14.2 | 312 |  |
|  |  | Never | 3.2 | 151 | 5.1 | 112 |  |
|  |  | Can't say | 2.0 | 97 | 5.2 | 114 |  |
|  | Swim or wade with at least one other person you know | Always | 39.0 | 1855 | 39.3 | 864 | χ2 = 0.20  (p=0.995) |
|  |  | Most of the time | 34.6 | 1649 | 34.1 | 750 |  |
|  |  | Sometimes | 22.6 | 1075 | 22.7 | 498 |  |
|  |  | Never | 2.6 | 124 | 2.7 | 59 |  |
|  |  | Can't say | 1.2 | 57 | 1.2 | 26 |  |
|  | Check surf conditions with a lifesaver, lifeguard or other authoritative source | Always | 23.9 | 1139 | 22.1 | 485 | χ2 = 27.68  (**p<0.001**) |
|  |  | Most of the time | 18.9 | 900 | 18.2 | 400 |  |
|  |  | Sometimes | 27.7 | 1318 | 26.4 | 580 |  |
|  |  | Never | 25.2 | 1198 | 26.2 | 575 |  |
|  |  | Can't say | 4.3 | 206 | 7.2 | 158 |  |
|  | Check for and obey safety signs posted on the beach | Always | 58.5 | 2783 | 59.0 | 1297 | χ2 = 22.55  (**p<0.001**) |
|  |  | Most of the time | 26.8 | 1276 | 25.0 | 550 |  |
|  |  | Sometimes | 11.5 | 549 | 10.7 | 236 |  |
|  |  | Never | 2.0 | 94 | 2.6 | 56 |  |
|  |  | Can't say | 1.2 | 59 | 2.6 | 58 |  |
|  | Look for the presence of rip currents in the area prior to entering the water | Always | 44.9 | 2138 | 50.1 | 1101 | χ2 = 23.37  (**p<0.001**) |
|  |  | Most of the time | 26.1 | 1243 | 23.5 | 516 |  |
|  |  | Sometimes | 18.3 | 872 | 14.8 | 326 |  |
|  |  | Never | 7.1 | 336 | 7.3 | 160 |  |
|  |  | Can't say | 3.6 | 171 | 4.3 | 94 |  |
|  | Avoid swimming or wading under the influence of alcohol \ drugs | Always | 68.8 | 3274 | 71.3 | 1567 | χ2 = 10.96  (**p=0.027**) |
|  |  | Most of the time | 18.5 | 882 | 18.0 | 396 |  |
|  |  | Sometimes | 8.4 | 401 | 6.4 | 140 |  |
|  |  | Never | 2.9 | 136 | 3.2 | 70 |  |
|  |  | Can't say | 1.4 | 68 | 1.2 | 26 |  |
|  | Follow the advice of the local lifesaver or lifeguard when you are on a patrolled beach | Always | 64.9 | 3091 | 66.4 | 1460 | χ2 = 101.40  (**p<0.001**) |
|  |  | Most of the time | 20.8 | 992 | 16.9 | 372 |  |
|  |  | Sometimes | 9.6 | 459 | 6.7 | 147 |  |
|  |  | Never | 1.9 | 93 | 3.1 | 69 |  |
|  |  | Can't say | 2.6 | 126 | 6.8 | 149 |  |
| **Surfing safety practices** | Surf with at least one other person you know | Always | 45.9 | 318 | 46.0 | 126 | χ2 = 1.79  (p=0.774) |
|  |  | Most of the time | 28.7 | 199 | 29.2 | 80 |  |
|  |  | Sometimes | 19.9 | 138 | 21.1 | 58 |  |
|  |  | Never | 4.4 | 30 | 2.7 | 7 |  |
|  |  | Can't say | 1.1 | 8 | 1.0 | 3 |  |
|  | Check surf conditions with a lifesaver, lifeguard or other authoritative source | Always | 33.1 | 229 | 31.2 | 86 | χ2 = 0.93  (p=0.920) |
|  |  | Most of the time | 22.7 | 158 | 25.1 | 69 |  |
|  |  | Sometimes | 26.4 | 183 | 25.6 | 70 |  |
|  |  | Never | 15.2 | 105 | 15.8 | 44 |  |
|  |  | Can't say | 2.6 | 18 | 2.3 | 6 |  |
|  |  | Selected | 4.8 | 31 | 5.5 | 14 |  |
| **Watercraft safety practices** | Use your watercraft with at least one other person you know | Always | 44.5 | 355 | 48.2 | 183 | χ2 = 2.65  (p=0.617) |
|  |  | Most of the time | 31.4 | 251 | 31.0 | 118 |  |
|  |  | Sometimes | 19.2 | 153 | 16.6 | 63 |  |
|  |  | Never | 3.9 | 31 | 2.9 | 11 |  |
|  |  | Can't say | 1.0 | 8 | 1.3 | 5 |  |
|  | Check conditions with a lifesaver, lifeguard or other authoritative source | Always | 32.7 | 261 | 28.1 | 107 | χ2 = 8.37  (p=0.079) |
|  |  | Most of the time | 24.2 | 193 | 21.0 | 80 |  |
|  |  | Sometimes | 23.8 | 190 | 27.1 | 103 |  |
|  |  | Never | 16.1 | 129 | 18.6 | 71 |  |
|  |  | Can't say | 3.1 | 24 | 5.2 | 20 |  |
|  | Follow all relevant laws and regulations applicable to watercraft | Always | 63.9 | 510 | 66.5 | 253 | χ2 = 3.12  (p=0.538) |
|  |  | Most of the time | 23.9 | 191 | 19.8 | 75 |  |
|  |  | Sometimes | 7.7 | 62 | 8.5 | 32 |  |
|  |  | Never | 1.6 | 13 | 1.4 | 5 |  |
|  |  | Can't say | 2.8 | 23 | 3.8 | 14 |  |
|  | Carry necessary safety equipment including EPIRBs (Emergency Position-Indicating Radio Beacons), radios or flares | Always | 27.7 | 221 | 35.6 | 135 | χ2 = 88.80  (p=0.066) |
|  |  | Most of the time | 13.6 | 108 | 11.8 | 45 |  |
|  |  | Sometimes | 14.6 | 117 | 14.8 | 56 |  |
|  |  | Never | 38.4 | 306 | 31.9 | 121 |  |
|  |  | Can't say | 5.8 | 46 | 5.9 | 22 |  |
|  | Avoid using your watercraft under the influence of alcohol/drugs | Always | 68.5 | 546 | 70.9 | 270 | χ2 = 6.26  (p=0.180) |
|  |  | Most of the time | 18.5 | 148 | 16.1 | 61 |  |
|  |  | Sometimes | 8.7 | 69 | 6.3 | 24 |  |
|  |  | Never | 3.2 | 26 | 5.6 | 21 |  |
|  |  | Can't say | 1.1 | 9 | 1.1 | 4 |  |
|  | Follow the advice of the local lifesaver or lifeguard when you are on a patrolled beach | Always | 56.9 | 454 | 52.4 | 199 | χ2 = 9.22  (p=0.056) |
|  |  | Most of the time | 22.0 | 176 | 19.3 | 73 |  |
|  |  | Sometimes | 10.1 | 80 | 13.3 | 51 |  |
|  |  | Never | 5.6 | 44 | 6.4 | 24 |  |
|  |  | Can't say | 5.4 | 43 | 8.6 | 33 |  |
|  | Wear a lifejacket or buoyancy aid | Always | 44.3 | 353 | 47.7 | 181 | χ2 = 2.43  (p=0.658) |
|  |  | Most of the time | 21.8 | 174 | 20.0 | 76 |  |
|  |  | Sometimes | 18.1 | 144 | 18.7 | 71 |  |
|  |  | Never | 14.3 | 114 | 11.8 | 45 |  |
|  |  | Can't say | 1.5 | 12 | 1.7 | 7 |  |
| **Watercraft safety equipment** | EPIRBs (Emergency Position-Indicating Radio Beacons) | Not Selected | 81.9 | 564 | 76.8 | 260 | χ2 = 3.48  (p=0.062) |
|  |  | Selected | 18.1 | 125 | 23.2 | 78 |  |
|  | Radios | Not Selected | 80.9 | 558 | 78.7 | 266 | χ2 = 0.67  (p=0.412) |
|  |  | Selected | 19.1 | 132 | 21.3 | 72 |  |
|  | Flares | Not Selected | 79.7 | 549 | 76.5 | 259 | χ2 = 1.26  (p=0.262) |
|  |  | Selected | 20.3 | 140 | 23.5 | 79 |  |
|  | Lifejacket | Not Selected | 24.2 | 167 | 23.3 | 79 | χ2 = 0.09  (p=0.769) |
|  |  | Selected | 75.8 | 523 | 76.7 | 259 |  |
|  | Buoyancy aid | Not Selected | 72.4 | 499 | 67.6 | 229 | χ2 = 2.50  (p=0.114) |
|  |  | Selected | 27.6 | 191 | 32.4 | 110 |  |
|  | Helmet | Not Selected | 87.6 | 604 | 90.9 | 308 | χ2 = 2.49  (p=0.115) |
|  |  | Selected | 12.4 | 86 | 9.1 | 31 |  |
|  | Shark deterrent device | Not Selected | 90.9 | 627 | 94.4 | 320 | χ2 = 3.61  (p=0.058) |
|  |  | Selected | 9.1 | 62 | 5.6 | 19 |  |
|  | Mobile phone | Not Selected | 46.0 | 259 | 35.7 | 91 | χ2 = 7.98  (**p=0.005**) |
|  |  | Selected | 54.0 | 303 | 64.3 | 165 |  |
|  | Other | Not Selected | 97.3 | 671 | 96.7 | 327 | χ2 = 0.20  (p=0.654) |
|  |  | Selected | 2.7 | 19 | 3.3 | 11 |  |
|  | Can’t say | Not Selected | 91.6 | 631 | 91.5 | 310 | χ2 = 0.01  (p=0.941) |
|  |  | Selected | 8.4 | 58 | 8.5 | 29 |  |
| **Rock-fishing safety practices** | Go rock fishing with at least one other person you know | Always | 49.1 | 184 | 57.7 | 162 | χ2 = 8.67  (p=0.070) |
|  |  | Most of the time | 25.8 | 96 | 17.3 | 49 |  |
|  |  | Sometimes | 17.7 | 66 | 19.6 | 55 |  |
|  |  | Never | 6.0 | 23 | 4.6 | 13 |  |
|  |  | Can't say | 1.4 | 5 | 0.8 | 2 |  |
|  | Check surf and weather conditions with a lifesaver, lifeguard or other authoritative source | Always | 35.9 | 134 | 38.2 | 108 | χ2 = 2.82  (p=0.589) |
|  |  | Most of the time | 26.3 | 98 | 20.9 | 59 |  |
|  |  | Sometimes | 19.3 | 72 | 19.6 | 55 |  |
|  |  | Never | 15.6 | 58 | 18.1 | 51 |  |
|  |  | Can't say | 2.9 | 11 | 3.2 | 9 |  |
|  | Check for and obey safety signs posted on the coast | Always | 49.2 | 184 | 59.3 | 167 | χ2 = 11.88  (**p=0.018**) |
|  |  | Most of the time | 26.3 | 98 | 23.2 | 65 |  |
|  |  | Sometimes | 16.4 | 61 | 8.8 | 25 |  |
|  |  | Never | 5.8 | 22 | 4.9 | 14 |  |
|  |  | Can't say | 2.4 | 9 | 3.8 | 11 |  |
|  | Wear appropriate clothing including non-slip footwear such as cleats | Always | 48.2 | 180 | 52.1 | 147 | χ2 = 13.53  (**p=0.009**) |
|  |  | Most of the time | 28.1 | 105 | 20.7 | 58 |  |
|  |  | Sometimes | 18.9 | 71 | 18.0 | 51 |  |
|  |  | Never | 3.2 | 12 | 8.6 | 24 |  |
|  |  | Can't say | 1.6 | 6 | 0.6 | 2 |  |
|  | Carry EPIRBs (Emergency Position-Indicating Radio Beacons), mobile phone or radio to alert others in an emergency | Always | 31.7 | 119 | 28.7 | 81 | χ2 = 11.56  (**p=0.021**) |
|  |  | Most of the time | 21.6 | 81 | 19.0 | 54 |  |
|  |  | Sometimes | 15.1 | 56 | 9.1 | 26 |  |
|  |  | Never | 28.4 | 106 | 39.8 | 112 |  |
|  |  | Can't say | 3.3 | 12 | 3.5 | 10 |  |
|  | Avoid rock fishing under the influence of alcohol/drugs | Always | 62.0 | 232 | 70.1 | 197 | χ2 = 8.19  (p=0.085) |
|  |  | Most of the time | 19.8 | 74 | 12.4 | 35 |  |
|  |  | Sometimes | 12.7 | 47 | 13.6 | 38 |  |
|  |  | Never | 3.4 | 13 | 2.9 | 8 |  |
|  |  | Can't say | 2.2 | 8 | 1.0 | 3 |  |
|  | Wear a lifejacket or buoyancy aid | Always | 26.2 | 98 | 20.1 | 57 | χ2 = 26.40  (**p<0.001**) |
|  |  | Most of the time | 22.6 | 85 | 11.4 | 32 |  |
|  |  | Sometimes | 16.4 | 62 | 19.5 | 55 |  |
|  |  | Never | 32.3 | 121 | 47.8 | 135 |  |
|  |  | Can't say | 2.4 | 9 | 1.1 | 3 |  |
| **Rock-fishing safety equipment** | EPIRBs (Emergency Position-Indicating Radio Beacons) | Not Selected | 84.1 | 305 | 89.6 | 235 | χ2 = 3.86  (**p=0.049**) |
|  |  | Selected | 15.9 | 57 | 10.4 | 27 |  |
|  | Radios | Not Selected | 79.8 | 289 | 85.8 | 225 | χ2 = 3.82  (p=0.051) |
|  |  | Selected | 20.2 | 73 | 14.2 | 37 |  |
|  | Mobile phone | Not Selected | 16.1 | 58 | 10.9 | 29 | χ2 = 3.17  (p=0.075) |
|  |  | Selected | 83.9 | 304 | 89.1 | 234 |  |
|  | Lifejacket | Not Selected | 60.0 | 218 | 74.3 | 195 | χ2 = 14.02  (**p<0.001**) |
|  |  | Selected | 40.0 | 145 | 25.7 | 67 |  |
|  | Buoyancy aid | Not Selected | 75.7 | 274 | 84.0 | 220 | χ2 = 6.32  (**p=0.012**) |
|  |  | Selected | 24.3 | 88 | 16.0 | 42 |  |
|  | Non-slip footwear/cleats | Not Selected | 34.5 | 125 | 32.5 | 85 | χ2 = 0.30  (p=0.586) |
|  |  | Selected | 65.5 | 237 | 67.5 | 177 |  |
|  | Other | Not Selected | 96.8 | 351 | 95.2 | 250 | χ2 = 1.07  (p=0.302) |
|  |  | Selected | 3.2 | 12 | 4.8 | 13 |  |
|  | Can't say | Not Selected | 97.8 | 354 | 98.1 | 257 | χ2 = 0.07  (p=0.795) |
|  |  | Selected | 2.2 | 8 | 1.9 | 5 |  |

*Notes:*

- *Significant chi-square analyses (p<0.05) are presented in bold.*
- **Other watercraft includes paddle craft, stand-up-paddle, paddle boarding, kite surfing and wind surfing.*
